# The Association between Clusterin Sialylation Degree and Levels of Oxidative–Antioxidant Balance Markers in Seminal Plasmas and Blood Sera of Male Partners with Abnormal Sperm Parameters

**DOI:** 10.3390/ijms231810598

**Published:** 2022-09-13

**Authors:** Ewa Janiszewska, Izabela Kokot, Agnieszka Kmieciak, Zuzanna Stelmasiak, Iwona Gilowska, Ricardo Faundez, Ewa Maria Kratz

**Affiliations:** 1 Department of Laboratory Diagnostics, Division of Laboratory Diagnostics, Faculty of Pharmacy, Wroclaw Medical University, Borowska Street 211A, 50-556 Wrocław, Poland; 2 Institute of Health Sciences, Collegium Salutis Humanae, University of Opole, Katowicka Street 68, 45-060 Opole, Poland or; 3 Clinical Center of Gynecology, Obstetrics and Neonatology in Opole, Reference Center for the Diagnosis and Treatment of Infertility, Reymonta Street 8, 45-066 Opole, Poland; 4 InviMed Fertility Clinics, Rakowiecka Street 36, 02-532 Warsaw, Poland

**Keywords:** clusterin, clusterin sialylation, markers of oxidative–antioxidant balance, male fertility disorders, male infertility diagnostics

## Abstract

Nearly 30% of infertility cases are caused by male factor. This study aimed at checking the associations between the sialylation degree of glycoprotein clusterin (CLU) and levels of oxidative–antioxidant balance markers in infertile men. Using lectin-ELISA with biotinylated lectins specific to α2,6-linked (*Sambucus nigra* agglutinin, SNA) and α2,3-linked (*Maackia amurensis* agglutinin, MAA) sialic acid (SA), the CLU sialylation in 132 seminal plasmas (SP) and 91 blood sera (BS) were analyzed. Oxidative–antioxidant status was measured by determining Sirtuin-3 (SIRT3), Sirtuin-5 (SIRT5), total antioxidant status (TAS), and ferric reducing antioxidant power (FRAP) levels. We indicate that multiple sperm disorders are associated with decreased expression of MAA-reactive SA in SP. Decreased SP SIRT3 concentrations may be associated with teratozoospermia and oligoasthenoteratozoospermia. ROC curve and cluster analysis revealed that SP relative reactivity of CLU glycans with MAA, the value of MAA/SNA ratio, and SIRT3 and SIRT5 concentrations may constitute an additional set of markers differentiating infertile oligoasthenoteratozoospermic patients (OAT) from normozoospermic (N), asthenoteratozoospermic (AT) and teratozoospermic (T). The multinomial logistic regression analysis confirmed the potential utility of SIRT3 determinations for differentiation between N and OAT groups as well as between N and T groups for SIRT3 and SIRT5. For BS, based on ROC curve and cluster analysis, relative reactivities of CLU glycans with SNA, MAA, SIRT3 and FRAP concentrations may be useful in the differentiation of normozoospermic patients from those with sperm disorders. The multinomial logistic regression analysis showed that the SNA relative reactivity with CLU glycans significantly differentiated the N group from AT, OAT and T groups, and FRAP concentrations significantly differed between N and AT groups, which additionally confirms the potential utility of these biomarkers in the differentiation of infertile patients with abnormal sperm parameters. The knowledge about associations between examined parameters may also influence future research aimed at seeking new male infertility therapies.

## 1. Introduction

Infertility is a disease of the reproductive system defined as the failure to achieve a clinical pregnancy after 12 months, or more, of regular unprotected sexual intercourse [1]. It is estimated that globally, nearly 30% of infertility cases are caused by male factor alone [2,3]. Routinely performed semen analysis points toward predominantly spermatozoa parameters (such as total count, motility, morphology, etc.); however, the early and sensitive biomarker of this disorder is still missing [4]. Almost 15% of infertile men are regarded as idiopathic with semen parameters within reference values [5,6]. Further investigations on molecular level, concerning this disease, are needed, and they may shed some new light on the problem of decreased male fertility issue, especially the idiopathic one.

Oxidative stress (OS) is defined as an imbalance between the physiological antioxidant mechanisms and reactive oxygen species (ROS) production [7]. In the male reproductive tract, a certain amount of superoxide anion is essential not only for proper capacitation, hyper-activation processes, and acrosome reaction but also for gamete fusion [8,9]. Nevertheless, many studies proved that ROS are one of the most common causes of decreased male fertility, affecting DNA (deoxyribonucleic acid) and RNA (ribonucleic acid) stability and lipid membrane peroxidation [10,11]. High ROS levels are often associated with decreased male fertility, confirmed also by sperm cells disorders, such as viability, motility and morphology [12,13]. In the case of excess ROS, seminal plasma releases a range of antioxidants that belong both to the non-enzymatic and enzymatic group [14,15].

Human ejaculate consists of abundant ions, lipids and glycoproteins taking part in a range of processes leading to the sperm cells’ production and maturation [16,17]. More than 6000 proteins and glycoproteins in human seminal plasma have been identified thus far [18], and clusterin (CLU, also known as Apolipoprotein J, ApoJ) was distinguished by Milardi et al. [19] as one of the main seminal plasma glycoproteins. This glycoprotein contains approximately 30% of carbohydrates and in its secretory isoform plays an important role in the male reproductive tract [20]. CLU is engaged in the semen liquefaction process via contact with eppin (epididymal protease inhibitor) present on the spermatozoa surface [21]. Seminal plasma CLU is liable for immune tolerance for male antigens in the female reproductive tract [22,23,24]. Taking into account the fact that CLU possesses at least four N-glycosylation sites as well as the importance of glycosylation in the sperm–oocyte interactions, the exploration of CLU glycans expression seems to be an important direction of study concerning male infertility issues [25,26]. Saraswat et al. [25] proposed over 40 complex type glyco-variants of CLU with terminal sialic acid (SA) or galactose. CLU glycans contained fucose, Le^x^/Le^a^, blood group H or Le^y^/Le^b^ oligosaccharide structures. Sialylation, attaching with sialic acids (group of chemical compounds represented by N-acetylneuraminic acid, Neu5Ac/NANA), is one of the most common posttranslational modifications. NANA affects the physiochemical and biological properties of glycoproteins, protects them against catabolic processes and takes part in immune recognition [27]. Rohne et al. [28] documented that the proteolytic cleavage of clusterin does not impact its chaperone activity, but only the completely glycosylated form of CLU is able to perform its chaperone activity, crucial for cell protection against destroying factors such as ionizing radiation, oxidative agents and others contributing to the oxidative–antioxidant imbalance [28]. The properties of CLU are similar to small heat shock proteins—it can bind unfolded proteins and block their aggregation regardless of the presence of ATP. This feature is particularly important in the DNA oxidation sperm damage context [29].

Sirtuins (SIRTs) are part of group of enzymes with activity of nicotinamide adenine dinucleotide (NAD^+^) deacetylases, existing in all cells’ compartments, taking part not only in damaged DNA reparation, but also in many processes connected with oxidative–antioxidant balance, cell cycle and metabolism regulation, differentiation, growth and apoptosis [30,31]. SIRT3 and SIRT5 concentrations are strictly associated with oxidative–antioxidant balance. SIRT3 occurs in the nucleus in its full form and is translocated to the mitochondria as a response for stress factors, i.a., mtDNA reparation and assurance of mitochondria integrity, but also for protecting the cell from apoptosis under conditions of oxidative stress [32]. SIRT5 may be located in the mitochondria, cytoplasm and nucleus, and it plays an important role in the apoptosis pathway, energy production, detoxification, cellular metabolism and oxidative stress regulation [31,33]. The mitochondrial form of this protein takes part in the protein modifications such as demalonylation, deacetylation, and desuccinylation [34,35]. It has been proven that cells transfected with SIRT5 have decreased ROS levels, suggesting that this enzyme suppresses excessive ROS production [36].

Taking into account the multidirectional role of CLU in human seminal plasma, including oxidative stress regulation via its chaperone activity and sperm cell protection against negative effects of oxidative stress, as well as the well-known biological role of glycoprotein glycosylation in a variety of processes in the human body, we decided to investigate the type and expression of sialic acid in seminal plasma and serum CLU glycans together with the expression of selected parameters of oxidative–antioxidant balance, and to check if there are any associations between the profile and degree of CLU sialylation and levels of oxidative stress markers such as seminal plasma and blood serum SIRT3 and SIRT5 concentrations. For antioxidant capacity assessment, total antioxidant status (TAS) and ferric reducing antioxidant power (FRAP) were selected. The potential use of the determinations of the selected parameters of oxidative–antioxidant balance in the diagnostics of male infertility as well as to expand knowledge about associations between examined parameters that may be helpful in developing new therapeutic strategies for male infertility, especially caused by multiple overlapping factors, were also in the sphere of our interests.

## 2. Results

The values of examined parameters are shown in Table 1.

### 2.1. Sialic Acids Expression in the Glycans of Serum and Seminal Plasma Clusterin

There were no significant differences between seminal plasma groups in relative reactivities of CLU glycans with SNA (median values: AT group: 0.203 AU, N group: 0.199 AU, OAT group: 0.184 AU, T group: 0.202 AU) (Table 1). Relative reactivities of CLU glycans with MAA in seminal plasmas were significantly lower in the OAT group (median value: 0.171 AU) in comparison to the N (median value: 0.376 AU, *p* = 0.005120), T (median value: 0.740 AU, *p* < 0.000001) and AT group (median value: 0.933 AU, *p* = 0.000003; Table 1).

Relative reactivities of serum CLU glycans with SNA of normozoospermic patients were significantly higher (median value: 0.881 AU) in comparison to the other examined groups: teratozoospermic (median value: 0.428 AU, *p* = 0.000009), asthenoteratozoospermic (median value: 0.412 AU, *p* = 0.000073) and oligoasthenoteratozoospermic (median value: 0.495 AU, *p* = 0.000123) (Table 1). There were no significant differences between serum groups in relative reactivities of CLU glycans with MAA (median values: AT group: 0.016 AU, N group: 0.081 AU, OAT group: 0.020 AU, T group: 0.035 AU) (Table 1).

The values of sialylation ratio (MAA/SNA) for seminal plasma samples were significantly lower in the OAT group (median value: 0.756) in comparison to the T (median value: 3.781, *p* = 0.000005) and AT group (median value: 4.455, *p* = 0.000054; Table 1). No significant differences between serum groups in the values of sialylation ratio were found, and the median values were following: 0.034 AU in AT group, 0.081 AU in N group, 0.036 AU in OAT group and 0.085 AU in T group (Table 1).

**Figure 1:**
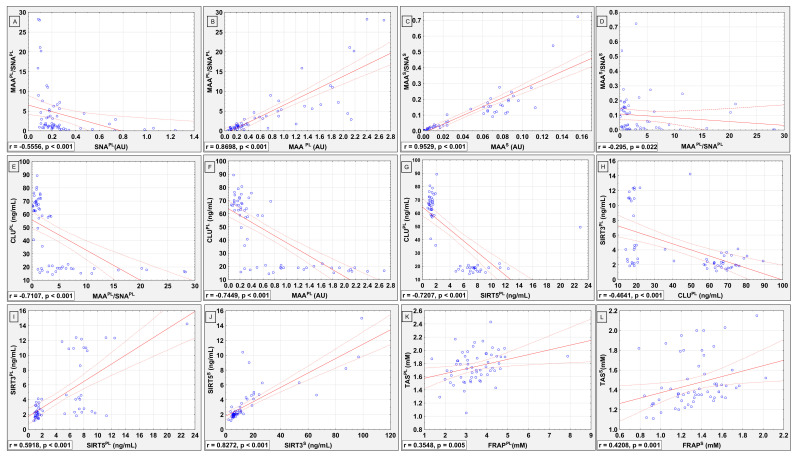
The correlations between selected parameters in seminal plasma (**A**,**B**,**E**–**I**,**K**) and blood serum (**C**,**J**,**L**) and comparison of MAA/SNA sialylation ratio between both biological fluids (**D**). MAA^PL^/SNA^PL^-seminal plasma sialylation ratio; SNA^PL^-relative reactivity of seminal plasma CLU glycans with *Sambucus nigra* agglutinin; MAA^PL^-relative reactivity of seminal plasma CLU glycans with *Maackia amurensis* agglutinin; MAA^S^/SNA^S^-serum sialylation ratio; MAA^S^-relative reactivity of serum CLU glycans with *Maackia amurensis* agglutinin; CLU^PL^-seminal plasma CLU concentration; SIRT5^PL^-seminal plasma SIRT5 concentration; SIRT3^PL^-seminal plasma SIRT3 concentration; SIRT5^S^-serum SIRT5 concentration; SIRT3^S^-serum SIRT3 concentration; TAS^PL^-seminal plasma total antioxidant status; FRAP^PL^-seminal plasma ferric reducing antioxidant power; TAS^S^-serum total antioxidant status; FRAP^S^-serum ferric reducing antioxidant power. The red dashed line points 95% of confidence interval. A two-tailed *p* value of less than 0.05 was considered significant.

The results of the correlation analysis between parameters investigated in seminal plasmas and sera are summarized in Figure 1A–L, which present only significant correlations. The comparison between seminal plasma MAA/SNA ratio and SNA relative reactivity with CLU glycans showed the presence of significant negative correlation between both parameters (r = −0.5556; *p* < 0.001; Figure 1A). The significant positive correlations between values of sialylation ratio and MAA relative reactivities with CLU glycans were found for seminal plasma and blood serum (r = 0.8698; *p* < 0.001 and r = 0.9529; *p* < 0.001, respectively; Figure 1B,C).

### 2.2. The Comparison of Sialylation Profile in the Seminal Plasma and Blood Serum

There were no significant correlations between both biological fluids analyzed by us in the relative expression of MAA- and SNA-reactive sialic acid in CLU glycans; however, a weak negative correlation between values of sialylation ratio (MAA/SNA) was found (r = −0.2950; *p* = 0.022, Figure 1D).

### 2.3. SIRTs Concentrations

Seminal plasma SIRT3 concentrations were significantly higher in the N group (median value: 9.35 ng/mL) in comparison to the T (median value: 2.64 ng/mL; *p* < 0.000001) and OAT group (median value: 2.11 ng/mL; *p* < 0.000001). Similar differences were observed between the AT group (median value: 10.90 ng/mL) vs. the T and OAT groups with significances of *p* = 0.000001 and *p* < 0.000001, respectively (Table 1). SIRT3 concentrations in sera were significantly lower in the N group (median value: 2.73 ng/mL) in comparison to the asthenoteratozoospermic (median value: 8.94 ng/mL; *p* = 0.000859) and oligoasthenoteratozoospermic (median value: 6.27 ng/mL; *p* = 0.002728) groups. The median value of serum SIRT3 concentrations in the teratozoospermic group was 5.93 ng/mL (Table 1).

Seminal plasma SIRT5 concentrations were significantly lower in the OAT group (median value: 1.34 ng/mL) in comparison to the N (median value: 7.28 ng/mL, *p* = 0.000019), T (median value: 6.89 ng/mL, *p* = 0.000423) and AT group (median value: 5.72 ng/mL, *p* = 0.000001; Table 1). No significant differences for serum SIRT5 concentrations were found. Median values of serum SIRT5 concentrations were following: 2.25 ng/mL in AT group, 2.01 ng/mL in N group, 2.02 ng/mL in OAT group and 2.05 ng/mL in T group (Table 1).

### 2.4. TAS Measurement

No significant differences for concentrations of seminal plasma and serum TAS were found (Table 1). Median values for seminal plasma TAS levels were the following: 1.79 mM in AT group, 1.71 mM in N group, 1.70 mM in OAT group and 1.72 mM in T group. Median values of serum TAS concentrations were: 1.37 mM in AT group, 1.54 mM in N group, 1.40 mM in OAT group and 1.38 mM in T group. (Table 1).

### 2.5. FRAP Determination

No significant differences in values of seminal plasma ferric reducing antioxidant capacity (FRAP) between analyzed groups were found (Table 1). Median seminal plasma FRAP values were as follow: 3.68 mM in AT group, 3.65 mM in N group, 3.04 mM in OAT group and 3.72 mM in T group. FRAP values in sera were significantly higher in the N group (median value: 1.49 mM) in comparison to the AT group (median value: 1.20 mM) with significancy of *p* = 0.005187. The median values of serum FRAP level in OAT and T groups were 1.33 and 1.38 mM, respectively (Table 1).

### 2.6. ROC Curves Analysis

The receiver operating characteristic curves were performed for all determined seminal plasma and serum parameters. Two ways of arranging the obtained results for ROC curves analysis were used, namely, the levels of each parameter were compared between: (1) two groups of patients distinguished on the basis of standard semen analysis (Table 2 and Table 3 for seminal plasma and blood serum, respectively); (2) each group of patients distinguished on the basis of seminogram analysis versus all other patients treated as one group (Supplementary Materials, Tables S2 and S3 for seminal plasma and blood serum, respectively). Based on the AUC, the clinical value of laboratory test can be defined as: 0–0.5—zero, 0.5–0.7—limited, 0.7–0.9—moderate and >0.9—high [37]. We used an AUC of ≥0.7, *p* < 0.05, as the criterion demonstrating the moderate clinical value of an examined parameter.

### 2.7. Cluster Analysis

During the preliminary cluster analyses of seminal plasma and blood serum data, we performed the analyses for all parameters in many variants, starting from the complete data (results not shown), which gave us the possibility to check for whether the parameters selected for the cluster analysis were a good choice. Finally, we decided that seminal plasma parameters chosen for the cluster analysis should comply with the following criteria: they differentiated the study groups with a two-tailed *p* value of less than 0.05 considered as significant (Table S1) and in the ROC curve analysis had moderate or high clinical value (AUC ≥ 0.700, *p* < 0.05) when the levels of given parameter were compared between two groups of patients distinguished on the basis of standard semen analysis (AUC ≥ 0.715, Table 2). To perform cluster analysis for seminal plasma, four parameters were selected: CLU relative reactivity with MAA, sialylation ratio (MAA/SNA) as well as SIRT3 and SIRT5 concentrations. Selected parameters also met the assumed selection criteria in the ROC curve analysis, in which each group of patients distinguished on the basis of seminogram analysis was compared with all other patients treated as one group (Table S2). The analysis was performed for 100 seminal plasma samples. The whole distance (100%) was considered at 35 value on the *x*-axis. At 91.6% distance, all samples could be regarded as homogenous formation (the value 32.1 on the *x*-axis). The first cluster was distinguished at 53.2% distance (the value 18.6 on the *x*-axis) and constitutes 11 samples. The second cluster could be distinguished at 52.8% distance (the value 18.5 on the *x*-axis) and is composed of two AT samples. The third cluster could be distinguished at 29.6% distance (the value 10.4 on the *x*-axis) and comprised 50% asthenoteratozoospermic samples (9 from 18 chosen for the analysis) and 40% normozoospermic samples (10 from 25 chosen for the analysis). Cluster no. 4 and 5 were distinguished at 29% and 26.4% distance, respectively (values on the *x*-axis: 10.2 and 9.2 on the *x*-axis, respectively). In cluster 4, there were 17 samples, and all of them were teratozoospermic (56.7% of T samples chosen for the analysis). Cluster 5 comprised 100% of analyzed OAT samples (Figure S1, Table S4).

In the case of serum, cluster analysis was performed for four parameters that differentiated the study groups with a two-tailed *p* value of less than 0.05 considered as significant (Table S1) and in the ROC curve analysis had moderate or high clinical value with AUC ≥0.701 when the levels of the given parameter were compared between two groups of patients distinguished on the basis of standard semen analysis: CLU relative reactivities with SNA and MAA, SIRT3 and FRAP concentrations (Table 3). Three out of four selected parameters (CLU relative reactivities with SNA and MAA, and SIRT3 concentrations) also met the assumed selection criteria when in the ROC curve analysis. Each group of patients distinguished on the basis of seminogram analysis was compared with all other patients treated as one group. Although this way of analyzing the ROC curves resulted in AUC for the FRAP concentration values being below 0.7 (Table S2), we decided to include this parameter in the cluster analysis. The analysis was performed for 89 serum samples. The whole distance (100%) was considered at 140 values on the *x*-axis. At 98.8% distance, all samples could be regarded as homogenous formation (the value 138.3 on the *x*-axis). The first cluster could be distinguished at 59.5% distance (the value 83.3 on the *x*-axis) and constitutes six samples. The second cluster was distinguished at 12.1% distance (the value 16.9 on the *x*-axis) and comprised 22 serum samples. The third cluster, distinguished at 4.6% distance (the value 6.4 on the *x*-axis), contained 60.7% of the OAT samples chosen for analysis (17 from 28 OAT samples) and 66.7% of T samples (20 from 30 T samples). The fourth cluster could be distinguished at 2.4% (the value 3.4 on the *x*-axis) distance and comprised 50% normozoospermic serum samples (8 form 16 N samples) (Figure S2, Table S5).

### 2.8. Relationships between the Group Classification of Patients vs. Seminal Plasma and Serum Parameters

Seminal plasma parameters: MAA^PL^, MAA^PL^/SNA^PL^, SIRT3^PL^ and SIRT5^PL^, taken into consideration during cluster analysis were also chosen as predictors in the multinomial logistic regression model. Due to the lack of representative fertile normozoospermic group of men, which is a limitation of our study, the infertile normozoospermic group was chosen as the reference group. In the case of seminal plasma, the Spearman’s rank correlation coefficient in correlation between MAA^PL^ and MAA^PL^/SNA^PL^ was 0.87, which means that the predictor collinearity assumption was not met here, and the results should be treated with caution. The model showed a significant effect of SIRT3^PL^ (OR = 0.236, *p* = 0.003 on the affiliation to the OAT or to the N group as well as the effect of SIRT3^PL^ (OR = 0.234, *p* < 0.001) and SIRT5^PL^ (OR = 2.008, *p* = 0.006; Table 4) on the affiliation to the T or N group.

Serum parameters: SNA^S^, MAA^S^, SIRT3^S^ and FRAP^S^ were chosen for the multinomial logistic regression model. Serum predictors, which were also chosen for the cluster analysis, did not demonstrate the collinearity (the pair-wise Spearman’s rank correlation coefficients were lower than 0.35). The model showed a significant effect of SNA^S^ (OR = 0.000, *p* = 0.003) on the affiliation to the AT or N group as well as on the affiliation to the OAT or N group (OR = 0.001, *p* = 0.007) and on the affiliation to the T or N group (OR = 0.000, *p* = 0.002). The model also showed a significant effect of FRAP^S^ on the affiliation to the AT or N group (OR = 0.016, *p* = 0.040; Table 5).

## 3. Discussion

### 3.1. Clusterin Concentration

Seminal plasma CLU concentration as well as serum levels of this glycoprotein were discussed in our previous study [38] in which we reported that the concentrations of seminal plasma clusterin were significantly higher in the OAT group in comparison to the normozoospermic, asthenoteratozoospermic and teratozoospermic groups (*p* = 0.000114, *p* = 0.000001 and *p* = 0.000003, respectively). Furthermore, in sera, the CLU concentrations were significantly lower in the normozoospermic group in comparison to the asthenoteratozoospermic (*p*  =  0.001718), oligoasthenoteratozoospermic (*p*  =  0.000318) and teratozoospermic (*p*  =  0.000183) groups [38]. We concluded that the differences in CLU expression in seminal plasma and blood sera may be caused by distinct location of CLU synthesis and by the biological role it plays depending on the part of the male organism [38]. To the best of our knowledge, there is lack of more recent data concerning this issue.

### 3.2. Sialic Acids Expression in the Glycans of Seminal Plasma and Blood Serum Clusterin

As far as we know, this is the first study assessing the semi-quantitative analysis of sialic acids expression in the glycans of seminal plasma and blood serum clusterin using lectin-ELISA assay. Lack of significant differences in relative reactivities of seminal plasma CLU glycans with SNA between studied groups may indicate that the expression of α2,6-linked SA is independent of sperm parameters assessed during routine semen analysis according to the WHO criteria used by us [39]. In our previous study on seminal plasma AGP (α1-acid glycoprotein) glycosylation [40], we also observed a lack of differences in AGP sialylation between seminal plasmas obtained from men with sperm abnormalities (astheno-, azoo-, oligozoo-, teratozoospermia, and mixed AT, OT, OAT) and normozoospermic infertile patients. As both CLU and AGP are known as acute phase proteins having anti-inflammatory properties, and they are synthesized locally within the male reproductive organs, the similarities in trend of their sialylation degree in human seminal plasma are not particularly surprising. However, these findings stand in contrast with our previous study, where the relative reactivities of seminal plasma glycoproteins with SNA were significantly lower in the asthenozoospermic group in comparison to the normozoospermic fertile and infertile group as well as to the oligozoospermic group; however, it should be taken into account that, now, we analyzed the sialylation of CLU, not the sialylation of the whole panel of seminal plasma glycoproteins [41].

Based on the results obtained, we can presume that multiple sperm disorders (containing more than one anomaly) may reflect the decrease in expression of α2,3-linked MAA-reactive SA in seminal plasma CLU glycans. Moreover, a strong negative correlation between seminal plasma CLU concentration and relative reactivity of CLU glycans with MAA (Figure 1F) suggest that high concentrations of seminal CLU are accompanied by lower expression of sialic acid α2,3-linked. We have found a similar relationship in our previous studies [40] focused on the analysis of AGP sialylation degree in infertile men with abnormal values of standard semen analysis parameters, in which it was observed that together with increasing AGP concentration, the expression of MAA-reactive sialic acid decreases, regardless of the presence or absence of sperm abnormalities. This may provide additional evidence that there are certain patterns of variability in the expression of glycans of acute phase glycoproteins.

The intravascular survival of most mammalian blood plasma glycoproteins is dependent upon the integrity of carbohydrate chains. Removal of the terminal sialic acid residues from glycoproteins results in their rapid transfer from circulation into the liver. The exposition of terminal galactose results shows that these proteins are rapidly transferred from the circulation into the hepatocellular lysosomes where they are catabolized [42]. The same mechanism of glycoprotein elimination applies to the serum CLU, but whether as well to the human semen CLU is an open question. To date, the role of sialic acids in CLU glycans has not been fully explored yet. There are no literature data concerning the specific role of SA in CLU glycans in cellular interactions, apoptosis and immune tolerance.

Analyzing the obtained results, we must also take into account other aspects that we are not able to verify and which may affect the degree of CLU sialylation. It has been proven that during permanent alcohol overdose, the CLU molecule is desialylated. Conversely, abstinence results in an increase in SA present on CLU glycans [43,44]. In 1995, Ghosh et al. [45] showed that chronic ethanol exposure can lead to the modification of Golgi apparatus membranes, which is a key cell compartment for clusterin sialylation. Loss of sialic acid by the clusterin molecule may result in a change in its molecular conformation, which may in turn affect its stability, antigenic expression or its receptors recognition. Hale et al. [46] in their study reported that long-term ethanol exposure significantly impairs sialylation, which is a key step in clusterin biosynthesis. Taking into account the above information, although we do not have information on the addictions of patients covered by our research, including increased alcohol consumption, we cannot exclude this factor as affecting the degree of CLU sialylation.

Kałuża et al. [47] determined the content of immunomodulatory glycoepitopes in seminal plasma glycoproteins. Mass spectrometry enabled for distinguishing several glycan types within CLU. Their results revealed significantly decreased relative reactivity with MAA of glycoprotein glycans in the band within the most matches for CLU in the oligoasthenozoospermic group in comparison to the normozoospermic, oligozoospermic as well as to the control group [47]. Our previous study concerning, i.a., sialylation profile of seminal plasma proteins, showed clearly that the relative reactivities of seminal plasma proteins with MAA were significantly lower in the oligozoospermic group compared to the infertile normozoospermic and asthenoteratozoospermic groups [41]. Quite apart from the different study groups, the discrepancies between our previous and present results are most probably caused by the fact that in the previous study, a mixture of seminal plasma glycoproteins was examined, and thus, their sialylation profile differs from those obtained in case of clusterin.

Current research also included the analysis of sialic acid expression in glycans of serum CLU, examined in context of decreased male fertility. The obtained results showed that the reactivity of serum CLU glycans with SNA increased in the infertile normozoospermic men in comparison to the other analyzed groups of patients. Hence, we can presume that any sperm disorders confirmed in the routine seminal analysis reflect the decrease in α2,6-linked sialic acid expression in the serum CLU glycans. Analyzing the profile and degree of CLU sialylation, both in the seminal plasma and in the serum, and the observed significant differences between the analyzed groups, the question arises of how the pattern and degree of blood serum CLU sialylation (and whether at all) is related to seminal plasma CLU sialic acid expression and if the analysis of serum CLU sialylation may be usable as an additional parameter associated with disorders of male gametes. There are no current literature data associated with particular CLU synthesis places. Rosenior et al. [48] in their study on rats proved that CLU is synthetized de novo in epithelial cells; however, there is no research concerning the issue of human seminal plasma CLU synthesis: is it derived only from male accessory glands or is the blood–testis barrier permeable for blood CLU synthetized in liver? Answers for such questions remain unknown.

Lack of significant correlations in sialic acids expression in CLU glycans between biological fluids compared by us may suggest the differences in mechanisms of seminal plasma and serum CLU sialylation, indicating organ-specific mechanisms and place of CLU synthesis associated with the biological functions it plays in each of body fluids analyzed in the present study; however, further research in this field is needed. Furthermore, a weak negative correlation between seminal plasma and serum CLU sialylation ratio MAA/SNA was found (Figure 1E). However, the relative reactivities of seminal plasma and serum CLU glycans with sialo-specific lectins are not associated with each other, but the proportion between sialic acids attached via α2,3 and α2,6 glycoside bound may have an impact on CLU structure and thus on its biological function, but it should be confirmed in an another study. It seems that although the intensity of processes of seminal plasma and blood serum CLU sialylation differ from each other, the proportions in the expression of MAA- and SNA-reactive sialic acids are somewhat similar. How important it could be from the point of view of CLU biological function and its utility in the diagnostics of male fertility is difficult to determine at this time, because additional tests are needed for deeper analysis of this issue.

### 3.3. SIRTs Concentrations

Our results concerning seminal plasma SIRT3 concentrations indicate that teratozoospermia and oligoasthenoteratozoospermia are associated with decreased levels of this enzyme. As far as we know, the literature data concerning seminal plasma SIRTs concentration are not so abundant. Nasiri et al. [49] assessed seminal plasma SIRTs concentrations within SIRT3 and other oxidative–antioxidant balance parameters in normozoospermic and asthenoteratozoospermic men. Results of the above study are in contrast to our findings because the authors reported that seminal plasma SIRT3 levels were significantly lower in the AT group in comparison to the N group [49]; however, none of the subjects from N and AT group analyzed by Nasiri et al. [49] declared proven fertility and/or fertility problems as it was for subjects from our groups of patients. Bello et al. [50] investigated the expression of mitochondrial sirtuins gene expression in semen of fertile and infertile (normozoospermic, asthenoteratozoospermic and oligosthenoteratozoospermic) men. The authors observed that SIRT3 gene expression was significantly reduced in infertile patients in comparison to fertile men; moreover, normozoospermic patients had significantly higher relative expression of SIRT3 mRNA in comparison to the other infertile groups [50], which allowed us to find some analogies with our findings for SIRT3 concentrations.

Based on the results regarding serum SIRT3 concentrations, we can presume that sperm abnormalities such as morphology, total sperm count and motility are associated with elevated serum SIRT3 levels. Although the observed lack of correlations in SIRT3 concentrations between studied body fluids, seminal plasmas and blood sera indicate that serum sirtuin-3 expression is independent on its seminal plasma expression, it is possible that systemic oxidative–antioxidant imbalance also affects male fertility disorders, which are manifested by abnormalities in the number, morphology and movement of sperm. As far as we know, there are no current literature data concerning SIRT3 concentrations analyzed in context of decreased male fertility, and thus, we cannot refer to the results obtained by other authors. Rato et al. [51] hypothesized that SIRT3 together with peroxisome proliferator-activated receptor γ coactivator 1α (PGC-1α) are associated with pre-diabetic state and oxidative stress. Results of their study proved that SIRT3 levels are significantly decreased in the testes of pre-diabetic rats, accompanied by decreased antioxidant capacity measured as FRAP levels [51]. Moreover, decreased SIRT3 concentrations promoted glycolysis in testes [51,52]. Taking into account the multidirectional role of testes in male fertility disorders, this information seems to be significant. Further investigations concerning the impact of testes glucose metabolism on sperm cells properties, together with SIRT3 concentrations and levels of other oxidative stress parameters, may shed some new light on the cause of male fertility problems.

Di Emidio et al. [53] suggests that despite that SIRT5 is located mainly in mitochondria, it probably serves as a regulator of redox homeostasis by initiating a multiple antioxidant response that activates not only mitochondria, but also other redox-active organelles. Taking into account this fact, lowered seminal plasma SIRT5 concentration in the OAT group in comparison to the other groups suggests that sperm abnormalities involving sperm count, motility and morphology are related to the increase in ROS formation, reflecting in the inadequate seminal plasma SIRT5 concentration. Bello et al. [50] also found that relative SIRT5 mRNA expression is significantly reduced in oligoasthenospermic and asthenospermic men compared to the fertile group. Lack of significant differences between analyzed groups in sera SIRT5 concentrations may indicate that oxidative–antioxidative balance disorders associated with the role of SIRT5 are confined to the male reproductive system; however, our hypothesis requires further studies.

Very strong and strong positive correlation between concentrations of SIRTs in serum (Figure 1I) as well as in seminal plasma analyzed by us (Figure 1J), respectively, may indicate their synergistic action in both analyzed body fluids. Furthermore, observed by us, lack of correlation in SIRT3 and SIRT5 levels between both examined biological fluids was most probably caused by the differences in mechanisms and intensity of their action in seminal plasma and blood serum. Taking into account that many enzymes, including sirtuins, can act both synergistically and antagonistically toward each other as well as toward other biologically active molecules, it is not surprising that in our study, a negative correlation between concentrations of seminal plasma CLU vs. SIRT3 and SIRT5 was found (Figure 1G,H). One of the possible explanations for the obtained results is the fact that both molecules, however, are associated with oxidative–antioxidant balance, but their modes of action are different, not excluding an antagonistic effect.

### 3.4. TAS Measurement

We found no significant differences between analyzed groups of patients in seminal plasma TAS levels that are in accordance with the results of our previous study concerning silent inflammatory markers examined in the context of oxidative stress [54]. The observed lack of significant differences between seminal plasma groups in TAS concentrations may be associated with group size, and/or, most likely, with the lack of normozoospermic control group composed from men with proven fertility, which is a limitation of our study. In addition, the information on patient BMI (body mass index), type of infertility, duration of infertility and diseases related to reduced fertility may be helpful in the interpretation of the obtained results. Moreover, a factor that may affect the level of the measured values of oxidative stress parameters is the intake of widely available antioxidant dietary supplements by infertile patients, which, unfortunately, we were not able to verify. Furthermore, exposure to pro-oxidative factors (e.g., in the work environment) can significantly affect the level of oxidative stress markers. Information on this subject would undoubtedly be helpful in interpreting the results we obtained. Khosrowbeygi and Zarghami [55] measured TAS levels in seminal plasmas of infertile asthenoteratozoospermic, asthenozoospermic, oligoasthenozoospermic patients and compared the obtained results with those for fertile normozoospermic men. The authors reported that seminal TAS concentrations were significantly lower in all infertile groups in comparison to fertile normozoospermic men [55]. Hosen et al. [56] investigated, i.a., seminal plasma TAS levels in fertile and infertile men and proved that seminal plasma TAS was significantly lower in the infertile patients. Similar trend was observed by Ozer et al. [57] who measured seminal plasma TAS levels in infertile teratozoospermic patients and fertile normozoospermic men and showed that TAS concentrations were significantly lower in the infertile group of patients. Fatima et al. [58] also investigated seminal plasma TAS concentrations in the seminal plasma of fertile normozoospermic and infertile asthenozoospermic and oligoasthenozoospermic patients. The authors also found lowered TAS concentrations in all infertile groups of men when compared with fertile subjects. It seems that seminal plasma total antioxidant status is decreased in infertile male patients, independent of the type of sperm disorders, which was also proven by Emokpae et al. [59] who revealed that seminal plasma TAS was significantly lower in the infertile group in comparison to the control group of fertile men.

Observed by us, a lack of significant correlations between TAS concentrations and sperm parameters such as total sperm count, motility and morphology confirm the abovementioned findings of other authors that TAS levels are independent of the type of sperm disorders (preliminary experiments, not published). Gumus et al. [60] investigated antioxidant parameters in the infertile azoospermic men in comparison to the fertile control group and concluded that seminal plasma TAS concentrations were significantly lower in the azoospermic group. Fingerova et al. [61] conducted the study using the same method as in our research, assessing both seminal plasma and serum TAS concentrations in two groups: a study group that was composed of infertile normozoospermic men and patients with combined sperm disorders, and control group of fertile men. Their findings were consistent with those mentioned before: seminal plasma TAS concentrations were significantly lower in the group of infertile patients when compared with normozoospermic fertile men [61].

There is a little literature data concerning serum TAS concentrations analyzed in the context of decreased male fertility. Our study revealed no significant differences in serum TAS concentrations between groups of infertile men. Results obtained by Fingerova et al. [61] showed a lack of significant differences in serum TAS levels between fertile and infertile men; however, the authors found strong positive correlations between seminal plasma and serum TAS concentrations [61], which stands in contrast to our results of correlations analysis (preliminary experiments, not published). Furthermore, Gumus et al. [60] found increased serum TAS levels in the azoospermic group of patients in comparison to the control group of fertile men. Further investigations are needed, concerning not only seminal plasma and serum TAS concentrations, but also the expression of other parameters being markers of systemic and/or local inflammatory condition, which may directly or indirectly influence TAS level.

### 3.5. FRAP Determination

To the best of our knowledge, this is the first study assessing and comparing FRAP levels in biological fluids such as seminal plasma and serum. Lack of significant differences in seminal plasma FRAP concentrations between studied groups may be associated with a relative small number of subjects in each of group. The study of Aktan et al. [62] assessing some oxidative stress parameters, i.a., FRAP levels in seminal plasmas of idiopathic infertile men and control group of fertile donors, also revealed no significant differences between these groups. Our previous study revealed that seminal plasma FRAP concentrations in azoospermic samples were significantly higher in comparison to the teratozoospermic and normozoospermic subjects [63]. Moreover, in our another study, we reported that seminal plasma FRAP levels were significantly higher in infertile normozoospermic and oligozoospermic patients in comparison to the fertile normozoospermic men [12]. The results obtained by Abdulrahman et al. [64] also documented that seminal plasma FRAP concentrations were significantly higher in the infertile normozoospermic group in comparison to the asthenozoospermic and oligoasthenozoospermic patients. Lack of a group of fertile normozoospermic men make such comparisons impossible in the present study. Pahune et al. [65] examined FRAP levels in seminal plasmas of asthenoteratozoospermic, oligoasthenoteratozoospermic and azoospermic patients and compared the obtained results with those of the fertile normozoospermic group, showing that seminal plasma FRAP concentrations were significantly lower in all infertile groups in comparison to the normozoospermic group and positively correlated with sperm concentration, motility and morphology [65]. These findings are similar to the research of Colagar et al. [66] who proved that seminal plasma FRAP was significantly lower in the AT group and OAT group in comparison to the fertile normozoospermic group; moreover, positive correlation between seminal plasma FRAP and sperm count, motility and morphology was found. In addition, Gholinezhad et al. [67] showed that seminal plasma FRAP was significantly lower in the infertile men and positively correlated with the sperm count and motility. In the present study, we did not find any significant correlations between semen parameters and the concentration of FRAP in seminal plasma (preliminary experiments, not published). Fazeli et al. [68] investigated, i.a., seminal plasma FRAP levels in the men with idiopathic infertility and compared the obtained results to those of fertile men, also proving that seminal plasma FRAP in the study group was significantly lower in comparison to fertile men. Nasiri et al. [49] found that seminal plasma FRAP concentrations in asthenoteratozoospermic group were significantly lower in comparison to the normozoospermic group of healthy men.

Based on the results of the current study concerning serum FRAP concentrations, we can presume that sperm motility abnormalities together with their morphology disorders reflect in the decrease in antioxidant capacity measured as FRAP concentration. Donatus et al. [69] assessed serum oxidative stress parameters including FRAP in infertile men (oligozoospermic, oligoasthenozoospermic and asthenozoospermic groups) and compared the obtained results to the control group of fertile men. They revealed that serum FRAP concentrations were significantly lower in all infertile groups [69].

Weak positive correlations were found between TAS and FRAP levels in both examined biological fluids (Figure 1K,L); however, Rao et al. [70] who also determined and compared these two oxidative stress parameters found no correlations between them in blood serum. Furthermore, when TAS was corrected for proteins, positive correlation was observed with FRAP [70].

### 3.6. ROC Curve Analysis

Based on the results of the ROC curve analysis carried out for each of the selected seminal plasma and blood serum parameters, we at first can propose the level of sensitivity and specificity as well as the value of cut-off point for each of them, simultaneously analyzing the potential clinical value each of the discussed parameters. A detailed presentation deserves the results of the ROC curve analysis for which the AUC value was equal to or higher than 0.700 (*p* < 0.05), which proves their moderate or high clinical value. We used two ways of arranging the obtained results for ROC curve analysis: (1) the values for a given parameter were compared between two groups of patients separated on the basis of standard semen analysis (Table 2 and Table 3 for seminal plasma and blood serum, respectively), and (2) between a given group of patients distinguished on the basis of standard semen analysis and other study groups gathered together and treated as one study group (Supplementary Materials, Tables S2 and S3 for seminal plasma and blood serum, respectively).

According to the first way of ROC curve analysis, we can presume that seminal plasma parameters such as relative reactivity of CLU glycans with MAA, MAA/SNA sialylation ratio, SIRT3 and SIRT5 concentrations may constitute additional markers differentiating patients with sperm disorders from those with normal sperm parameters (Table 2).

In this study, relative reactivities of seminal plasma CLU glycans with MAA have a moderate clinical value and enabled differentiation of the OAT group from (1) normozoospermic men with sensitivity and specificity of 79.3% and 72.4%, respectively (proposed cut off point: 0.224 AU, AUC = 0.715), (2) teratozoospermic group with sensitivity and specificity of 79.3% and 91.2%, respectively (proposed cut off point: 0.224 AU, AUC = 0.890) and (3) asthenoteratozoospermic group with sensitivity and specificity of 75.9% and 94.4%, respectively (proposed cut off point: 0.214 AU, AUC = 0.883). Based on this, we can conclude that altered expression of α2,3-linked sialic acid in seminal plasma CLU glycans is strictly associated with mixed sperm disorders such as decreased total sperm count, motility and sperm morphology abnormalities. The above observations were also confirmed by the results of the ROC curve analysis, in which CLU reactivity with MAA also has a moderate clinical value in differentiation of the OAT group from the other groups of patients treated as one group (proposed cut off point: 0.214 AU, AUC = 0.826, sensitivity 75.9%, specificity 85.2%).

The sialylation ratio has a moderate clinical value and enabled differentiation of the OAT group from the teratozoospermic patients with sensitivity and specificity of 89.7% and 67.6%, respectively (proposed cut off point: 2.033, AUC = 0.836) and from the asthenoteratozoospermic patients with sensitivity and specificity of 55.2% and 100%, respectively (proposed cut off point: 0.824, AUC = 0.837). The MAA/SNA ratio also has a moderate clinical value when the OAT group was compared with the other patients treated as one group (the proposed cut off point was 2.033, AUC = 0.777, sensitivity 89.7%, specificity 55.6%).

The observed differences between clinical values of relative reactivities of CLU glycans with the MAA and MAA/SNA ratio may suggest that the expression of α2,3-linked SA seems to be a more suitable marker for seminal plasma group differentiation than the ratio of α2,3-linked to α2,6-linked sialic acid. Furthermore, taking into account the shown differences in MAA relative reactivity with CLU glycans as well as in the level of MAA/SNA ratio between OAT patients vs. other analyzed groups taken together, both parameters seem to be usable for differentiation of the OAT group from AT, N and T groups, but the values of relative reactivities of CLU glycans with MAA seems to be more strongly associated with lowered sperm count.

Seminal plasma SIRT3 concentrations, when compared between two groups of patients distinguished on the basis of standard semen analysis, have a high clinical value and enabled differentiation of the OAT group from the normozoospermic group with sensitivity and specificity of 82.1% and 94.6%, respectively (proposed cut off point: 2.5 ng/mL, AUC = 0.951) and from the asthenoteratozoospermic group with sensitivity and specificity of 100% and 81.8%, respectively (proposed cut off point: 7.985 ng/mL, AUC = 0.948). This parameter also enabled the differentiation of the AT group from the teratozoospermic group with sensitivity and specificity of 81.8% and 100%, respectively (proposed cut of point: 8.604 ng/mL, AUC = 0.906). Seminal plasma SIRT3 has a moderate clinical value in the differentiation of the T group from the normozoospermic group with sensitivity and specificity of 100% and 75.7%, respectively (proposed cut off point: 4.645 ng/mL, AUC = 0.887). Based on the obtained results, we can presume that seminal plasma SIRT3 concentration may be used as an additional marker helpful in the discrimination between patients with one or two sperm disorders as well as with two and three sperm disorders found in routine semen analysis. The above observation was confirmed by the results of ROC curve analysis in which each of the seminal plasma groups (AT, N, OAT and T) was compared with the rest of the groups together. SIRT3 concentration has a moderate clinical value (AUC: 0.720–0.847) with sensitivity 75.7–100% and specificity of 54–80.9%. SIRT3 is the only seminal plasma parameter for which the mean value of AUC has a moderate clinical value (AUC = 0.786).

Seminal plasma SIRT5 has a moderate clinical value and enabled differentiation of the OAT group from normozoospermic men with sensitivity and specificity of 96.4% and 73%, respectively (proposed cut off point: 2.084 ng/mL, AUC = 0.812) and from the teratozoospermic group with sensitivity and specificity of 96.4% and 69.4%, respectively (proposed cut off point: 2.084 ng/mL, AUC = 0.759), but seminal plasma SIRT5 concentration has a high clinical value when distinguishing the OAT group from the asthenoteratozoospermic group with sensitivity and specificity of 96.4% and 81.8%, respectively (proposed cut off point: 2.084 ng/mL, AUC = 0.916). Moreover, seminal plasma SIRT5 concentration has a moderate clinical value and enabled differentiation of the OAT group from other samples treated as one group, with sensitivity and specificity of 96.4% and 73.7%, respectively (proposed cut off point: 2.084 ng/mL, AUC = 0.816).

ROC curve analysis of serum parameters showed that relative reactivities of CLU glycans with SNA and MAA, together with SIRT3 and FRAP concentrations, may be useful in the differentiation of infertile men with abnormal as well as normal semen parameters (Table 3).

Relative reactivity of serum CLU glycans with SNA, when compared between two groups of patients separated on the basis of standard semen analysis, has a moderate clinical value and enabled differentiation of N patients from: (1) asthenoteratozoospermic subjects with sensitivity and specificity of 86.7% and 81.3%, respectively (proposed cut off point: 0.609 AU, AUC = 0.892), (2) oligoasthenoteratozoospermic patients with sensitivity and specificity of 88.9% and 68.8%, respectively (proposed cut off point: 0.694 AU, AUC = 0.837), and (3) teratozoospermic patients with sensitivity and specificity of 87.1% and 81.3%, respectively (proposed cut off point: 0.591 AU, AUC = 0.872). Relative reactivity of serum CLU glycans with MAA has a moderate clinical value and enabled differentiation of the N group from the asthenoteratozoospermic group with sensitivity and specificity of 60% and 87.5%, respectively (proposed cut off point: 0.023 AU, AUC = 0.756) and from the teratozoospermic group with sensitivity and specificity of 51.6% and 87.5%, respectively (proposed cut off point: 0.035 AU, AUC = 0.716). The moderate clinical values were also observed in the ROC curve analysis, when CLU reactivity with SNA and MAA was compared between the N group and the other groups of patients gathered together (for SNA: proposed cut off point: 0.612 AU, AUC = 0.863, sensitivity 83.1%, specificity 82.2%; for MAA: proposed cut off point: 0.036 AU, AUC = 0.700, sensitivity 87.5%, specificity 54.8%).

Serum SIRT3 concentration enabled differentiation of the AT group from the normozoospermic patients with sensitivity and specificity of 100% and 61.1%, respectively (proposed cut off point: 4.979 ng/mL, AUC = 0.830) and from teratozoospermic patients with sensitivity and specificity of 93.3% and 51.6%, respectively (proposed cut off point: 6.044 ng/mL, AUC = 0.740). This parameter also enabled differentiation of the OAT group from normozoospermic patients with sensitivity and specificity of 100% and 61.1%, respectively (proposed cut off point: 5 ng/mL, AUC = 0.761). Serum SIRT3 concentration also has a moderate clinical value in differentiation of asthenoteratozoospermic and normozoospermic groups from other samples treated as one group, with sensitivity and specificity of 93.3% and 52.6%; 61.1% and 94.5%, respectively (proposed cut off points: 6.044 ng/mL, AUC = 0.714 and 3.608 ng/mL, AUC = 0.749, respectively).

Serum FRAP concentration enabled, with a moderate clinical value, differentiation of the AT group from the normozoospermic group with sensitivity and specificity of 80% and 77.8%, respectively (proposed cut off point: 1.352 mM, AUC = 0.780) and from the teratozoospermic group with sensitivity and specificity of 60% and 77.4%, respectively (proposed cut off point: 1.208 mM, AUC = 0.701). For this serum parameter, the results of the ROC curve analysis in which one group of patients is distinguished on the basis of standard semen analysis was compared with other study groups gathered together and treated as one study group, which did not confirm its moderate or high clinical value.

The results of the ROC curves analysis discussed by us indicate those of the proposed parameters that may be usable in the diagnosis of certain types of male infertility and may constitute the basis for further research, extended with additional parameters of the oxidative–antioxidant balance. It would also be advisable to include additional data on patients in future studies (e.g., BMI, comorbidities, duration of infertility) and to relate the results obtained for patients with fertility problems, especially for those with idiopathic infertility, to the group of healthy, normozoospermic men with proven fertility.

### 3.7. Cluster Analysis

On the basis of ROC curve analysis results, seminal plasma and serum parameters with AUC equal to or greater than 0.715 and 0.701, respectively, and simultaneously differentiated studied groups of patients, were selected for cluster analysis. Seminal plasma cluster analysis showed that the following parameters: relative reactivities of CLU glycans with MAA, MAA/SNA sialylation ratio, SIRT3 and SIRT5 concentrations, may be useful in the differentiation of the OAT group from patients with normal semen parameters, as well as from subjects with decreased sperm motility and/or abnormal morphology. Furthermore, serum parameters selected for cluster analysis such as relative reactivities of CLU glycans with SNA and MAA together with SIRT3 and FRAP concentrations may be taken into account as a promising additional set of markers helpful in the differentiation of normozoospermic patients from those with sperm disorders manifested as teratozoospermia, asthenoteratozoospermia and oligoasthenoteratozoospermia. The results of cluster analysis were in accordance with the results of the Wilcoxon test and ROC curve analysis, indicating that seminal plasma and blood serum parameters selected by us as a set of additional biomarkers of male infertility associated with sperm disorders are worth taking into consideration as a base for construction the diagnostic algorithms of male infertility associated with sperm abnormalities.

### 3.8. The Relationships between the Selected Markers and Classifying Patients into Study Groups

To assess the diagnostic utility the set of parameters proposed by us as the additional markers of male infertility linked with sperm disorders, multinomial logistic regression analysis was performed. The normozoospermic group was defined as a reference group. A *p* value of less than 0.05 was considered significant. In the case of seminal plasma, the results of multinomial regression analysis indicated the significant differences between the N and OAT groups for SIRT3 only, as well as between the N and T groups for SIRT3 and SIRT5 (Table 4). For serum parameters selected for the multinomial regression model, the SNA relative reactivity with CLU glycans was the parameter that significantly differentiated the normozoospermic group from the AT, OAT and T groups. Additionally, FRAP concentrations significantly differed between the N and AT groups (Table 5). The results obtained in this analysis additionally confirmed the potential diagnostic utility of some of the biomarkers we have selected for differentiation between the infertile normozoospermic group of patients and other infertile men.

## 4. Materials and Methods

### 4.1. Patient Samples

The seminal plasma and serum samples were collected between 2019 and 2020 from 132 infertile male patients visiting the Clinical Center of Gynecology, Obstetrics and Neonatology in Opole (Poland) and Fertility Clinics InviMed in Warsaw (Poland). Each patient gave informed consent for this study. Our study was conducted according to the guidelines of the Helsinki II declaration, and the protocol was approved by the Bioethics Human Research Committee of Wroclaw Medical University (no. KB 549/2019 and no. KB 707/2019).

The ejaculates were collected via masturbation into sterile containers after 3–5 days of sexual abstinence. After liquefaction (maximum 60 min at 37 °C), standard semen analysis was carried out according to WHO 2010 directives [39] (i.a., semen volume, pH and sperm viability), and supplemented by using computer-assisted sperm analysis (total sperm count in ejaculate, sperm concentration, total motility, progressive motility, and morphology), SCA Motility and Concentration, software version 6.5.0.5. (Microptic SL, Barcelona, Spain). All input data in this method were consistent with current WHO semen analysis recommendations [39]. Next, the ejaculates were centrifuged at 3500× *g* for 10 min at room temperature. Serum samples were obtained by peripheral blood collection and after coagulation centrifuged at 2000× *g* for 10 min at room temperature. All samples were then aliquoted and frozen at −86 °C until use.

Based on the results of standard semen analysis (sperm concentration, progressive motility, morphology of spermatozoa), seminal samples (n = 132) were divided into groups: asthenoteratozoospermic (AT, n = 22; <32% of sperm demonstrated progressive motility and lower than 4% of spermatozoa had normal morphology, the median age: 34 years (IQR 31–36)), normozoospermic (N, n = 43, normal values of ejaculate parameters, the median age: 32 years (IQR 24–49)), oligoasthenoteratozoospermic (OAT, n = 29; sperm count lower than 15 × 106 mL^−1^, <32% of sperm demonstrated progressive motility and lower than 4% of spermatozoa had normal morphology, the median age: 32 years (IQR 30–35)) and teratozoospermic (T, n = 38; lower than 4% of spermatozoa had normal morphology, the median age: 33 years (IQR 28–36)). Corresponding blood serum samples (n = 91) were divided into asthenoteratozoospermic (AT, n = 15), normozoospermic (N, n = 18), oligoasthenoteratozoospermic (OAT, n = 27) and teratozoospermic (T, n = 31) groups. In the normozoospermic ejaculates, the concentration of spermatozoa was higher than 15 × 10^6^ mL^−1^, and >4% of sperm exhibited normal morphology, with a total motility of ≥40% or progressive motility ≥32% (0.5 h after ejaculation). None of the seminal samples were leukospermic and/or infected by bacteria, and none of the serum samples were hemolyzed.

### 4.2. Methods

#### 4.2.1. Clusterin Concentration

Seminal plasma and blood serum CLU concentrations were determined using commercial enzyme-linked immunosorbent assay (ELISA): Human Clusterin ELISA kit from Bioassay Technology Laboratory (catalog no. E1189Hu; Shanghai, China) and Human Clusterin Elisa Kit from Invitrogen (Thermo Fisher Scientific, catalog no. EHCLU; Frederick, MD, USA), respectively, as described by us previously [38]. The determination of the CLU concentration was the basis for calculation of the constant amount of glycoprotein in 100 µL of solution that was applied to the well of ELISA plate for lectin-ELISA analysis of CLU sialylation (see “Sample dilution” in Section 4.2.3). All tests were performed without any modifications, according to the manufacturer instructions. The intra-assay and inter-assay coefficients of variations (CV%) for both tests were defined by the manufacturers and were also mentioned in our previous article [38].

#### 4.2.2. Determination of Sialic Acid Expression in the Seminal Plasma and Blood Serum Clusterin Glycans

Two biotinylated SA-specific lectins: *Sambucus nigra* agglutinin (SNA, catalog number B-1305, Vector Laboratories Inc., Burlingame, CA, USA) and *Maackia amurensis* agglutinin (MAA, catalog number B-1265, Vector Laboratories Inc., Burlingame, CA, USA) were used to determine sialic acid expression in the lectin-ELISA procedure according to Kratz et al. [26] with modifications described below. The specificity of lectins is following: SNA detects terminal sialic acids α2,6-linked to galactose (Gal) of antennary part of glycan, whereas MAA is specific to SA α2,3-linked to antennary Gal [71].

#### 4.2.3. Lectin-ELISA Procedure

##### ELISA-Plate Coating

The wells of ELISA plate (Nunc MaxiSorp, Thermo Fisher Scientific, Glostrup, Denmark) were coated by goat anti-human clusterin polyclonal antibodies (Invitrogen, Thermo Fisher Scientific, catalog no. PA1-26903; Rockford, IL, USA). The antibodies were diluted in 10 mM TBS, pH = 8.5. For SNA and MAA, the dilutions of 1:10,000 and 1:5000, respectively, were established. After 2 h incubation at 37 °C, the plate was washed three times by the same buffer. Free binding sites were blocked by 10 mM TBS, 0.1% Tween20, 1% BSA, pH = 7.5 (blocking buffer); next, the plate was incubated for 2 h at 37 °C and then stored at 4 °C overnight.

##### Sample Dilution

Seminal plasma and sera samples were diluted in 10 mM TBS 0.1% Tween20 buffer was used to obtain proper CLU amount in each well: 1 ng CLU/100 µL for seminal plasma and 50 ng CLU/100 µL form serum samples. The amounts of seminal plasma and serum CLU for lectin-ELISA were calculated basing on the CLU concentrations determined by us previously [38]. After application of the proper CLU amount in each well, the ELISA plate was incubated at 37 °C for two hours with gentle shaking. All samples were analyzed in duplicate to minimize imprecision. To each lectin–ELISA experiment, two pairs of blank were added. They contained all reagents, but instead of patients’ samples, 10 mM TBS, 0.1% Tween20, pH = 7.5 (washing buffer) was used. After each next step of lectin–ELISA, the wells were washed using washing buffer.

##### Lectin-SA Interactions

In the next step, the biotinylated lectins SNA and MAA, specific to α2,6-linked and α2,3-linked sialic acid, respectively, were used. The dilutions of lectin used were established in the series of initial experiments using 10 mM TBS containing 1 mM CaCl_2_, 1 mM MgCl_2_ × 6H_2_O, 1 mM MnCl_2_ × 4H_2_O, 1% BSA and 0.1% Tween20, pH = 7.5. *Sambucus nigra* agglutinin was diluted 1:2000, whereas *Maackia amurensis* agglutinin was diluted 1:250. Then, plates were incubated one hour at 37 °C with gentle shaking.

##### The Detection Clusterin–Lectin Complexes

To detect the clusterin–lectin complexes, ExtrAvidin alkaline phosphatase labeled (Sigma-Aldrich, catalog no. E2636; Saint Louis, MO, USA), diluted 1:10,000 in the washing buffer, was used. Next, plates were incubated for one hour at 37 °C, and then, the color reaction with disodium para-nitrophenyl phosphate was induced. The absorbances were measured with Mindray MR-96A Microplate Reader (Mindray Bio-Medical Electronics, Shenzen, China) at 405 nm with a reference filter 630 nm. The relative reactivities of sialic acid with lectins were expressed in absorbance units (AU), after subtracting the absorbances of the blank samples. Sialylation ratio (MAA/SNA) was calculated as the ratio between SA MAA reactivity to SNA reactivity.

#### 4.2.4. SIRTs Concentration

SIRT3 and SIRT5 concentrations were estimated using Human Sirtuin3 ELISA Kit (catalog number: E2559Hu, Bioassay Technology Laboratory, Shanghai, China) and Human Sirtuin-5 ELISA Kit (catalog number: E2561Hu, Bioassay Technology Laboratory, Shanghai, China), respectively. All tests were performed following manufacturer instructions, without any modifications. The intra- and inter-assay precisions, expressed as CV%, were determined by manufacturer, and for both tests, the intra-assay CV ranged <8% and the inter-assay was <10%.

#### 4.2.5. Total Antioxidant Capacity Assessment

Total antioxidant capacity (TAC) was expressed as measurement of total antioxidant status (TAS) and ferric reducing antioxidant power (FRAP), and their measurement procedures are described below.

##### Total Antioxidant Status Measurement

The levels of total antioxidant status for total antioxidant capacity of patient samples were performed on the autoanalyzer Konelab20i (Thermo Scientific, Vantaa, Finland) using TAS Randox reagents (TAS, catalog number NX2332, Crumlin, UK) according to manufacturer instructions, as described by us previously [72]. In short, the stable blue-green radical cation is a product of incubation (2,2′-Azino-di-[3-ethylbenzthiazoline sulphonate] with a peroxidase (metmyoglobin) and H_2_O_2_. Antioxidants from the patient’s sample suppress the color production proportionally to their concentration. The measurements were performed at 600 nm. The calibration curve with linearity up to 2.50 mM of Trolox standard was constructed, and thus, results were expressed as Trolox equivalents in mM.

##### Ferric Reducing Antioxidant Power Assessment

Ferric reducing antioxidant power (FRAP) was measured using spectrophotometric method according to the Benzie et al. [73]. Reduction of Fe^3+^ to Fe^2+^ at low pH leads to formation of a Fe^2+^–tripyridyltriazine complex. For these reactions, 500 µL freshly prepared FRAP reagent (25 mL of 300 mM acetate buffer (pH 3.6)), 2.5 mL of 10 mM 2,4,6-tripyridyl-s-triazine (TPTZ; Sigma-Aldrich, St. Louis, MO, USA) in 40 mM HCl and 2.5 mL of 20 mM aqueous solution of FeCl_3_ × 6H_2_O (CHEMPUR, Piekary Śląskie, Poland) was mixed with 100 µL of a 1:20 diluted seminal plasma sample and 1:10 diluted serum sample (diluted in distilled water). The reaction mixture was incubated at 37 °C for 5 min and then centrifuged for 10 min at 4000 rpm. The absorbance was measured at 593 nm in a VWR, UV-6300PC UV-VIS Spectrophotometer (Avantor, Radnor Township, PA, USA). The absorbance of the samples was read against blank samples containing the FRAP reagent instead of biological material. A calibration curve was prepared basing on the aqueous FeSO_4_ solution, within concentration ranges from 0.025 to 0.05 mM Fe^2+^. The seminal plasma and serum samples with a known FRAP concentration were used as our internal controls. All measurements were performed in duplicate.

#### 4.2.6. Statistical Analysis

Statistica 13.3PL software (StatSoft Inc., Tulsa, OK, USA) was used to perform the statistical analysis. To analyze the normality of distribution for values of all parameters investigated, the Shapiro–Wilks’ test was used. Values obtained for relative reactivities with lectins were presented as median with interquartile range (Q1–Q3). As the values of examined parameters were not drawn with normal distribution, to compare the relative reactivities of CLU glycans with sialo-specific lectins SNA and MAA, as well as the concentrations of oxidative stress parameters (SIRTs, TAS, FRAP) between examined groups, the nonparametric Mann–Whitney U test with Bonferroni correction was used. Taking into account the significance of pairwise differences, a *p* value of less than 0.008 was accepted as the level of significance. The relationships between values of determined parameters in seminal plasma and in sera, as well as between these both biological fluids, were checked by Spearman’s rank correlation. The receiver operating characteristic (ROC) curves were used to determine the diagnostic significance of relative reactivities of CLU glycans with sialo-specific lectins, and levels of SIRT3, SIRT5, TAS and FRAP, which were aimed at selecting parameters of the examined biological fluids that will have moderate or high clinical value for differentiation of patients with various variants of sperm disorders, and then, they were used to cluster analysis. Cluster analysis was performed for seminal plasma and serum samples, separately for both these body fluids, in which all parameters selected on the basis of the results of the ROC curve analysis were determined: the results were presented as a dendrogram that represents complete linkage clustering, in agglomerative hierarchical clustering. The data were not normalized before analysis, and all obtained values for each of the parameters tested in this analysis were absolute numbers. First, all examined subjects were gathered in one cluster, and then, the patients were clustered into the next clusters in which the subjects were more similar to each other. Patients that performed parallel in terms of the values of all the analyzed traits were grouped together, and a separate cluster was formed for those with different values. In conclusion, the smaller distance of separation means greater similarities in subject characteristics. The similarities between samples were calculated using a Euclidean metric on the original data points, with no reference to the clinical status of the subjects. The selection criteria were based on the results of Mann–Whitney U test with *p* values less than 0.05 considered as significant (Supplementary Materials, Table S1).

To check the diagnostic utility of the set of male infertility biomarkers proposed by us, multinomial logistic regression analysis was performed, in which the N group was defined as a reference group. The collinearity of the predictors was checked with the use of pair-wise Spearman’s rank correlation coefficients. A *p* value less than 0.05 was considered as significant. Results of the multinomial logistic regression for seminal plasma and blood serum are shown in Table 4 and Table 5, respectively.

## 5. Conclusions

The differences observed between groups of patients with abnormal sperm parameters and normozoospermic infertile men in relative reactivities of seminal plasma and serum CLU glycans with sialo-specific lectins MAA and SNA, respectively, may indicate that the alterations in CLU sialylation degree may be linked with improper sperm production and maturation, which as a consequence may lead to decreased fertility of men at the reproductive age. Lack of significant differences between analyzed groups of patients in relative reactivities of seminal plasma CLU glycans with SNA suggests that the expression of α2,6-linked SA in CLU is independent of sperm parameters. Furthermore, our results showed that any sperm disorders reflect the decrease in α2,6-linked SA in serum CLU glycans. Based on the results of our study, this serum parameter differentiates normozoospermic infertile men from patients with abnormal sperm parameters with high sensitivity and specificity and thus may be taken into account as an additional male infertility biomarker. However, additional confirmation of these hypotheses is needed in further studies carried out in groups with a larger number of patients, including the group of normozoospermic men of proven fertility. There is a possibility of different mechanisms of clusterin sialylation in both biological fluids analyzed by us, which may be indicated by the lack of significant correlations in relative reactivities of CLU glycans with SNA and MAA between blood serum and seminal plasma samples. We also observed that the degree of expression of MAA-reactive α2,3-linked sialic acid in the seminal plasma CLU differentiate the OAT group of men with reduced sperm count, abnormal motility and morphology of sperm from the other examined groups of patients, and this parameter has moderate clinical value. Taking into consideration the above observations, the level of seminal plasma CLU reactivity with MAA seems to be associated with sperm count disorders. Moreover, the values of seminal plasma MAA/SNA ratio also significantly decreased in patients with lowered sperm count when compared to patients with other sperm abnormalities.

The results of our study support the thesis that oxidative stress plays an important role in the male infertility issue. Sperm cells’ alterations in their morphology, motility or total count may reflect in the increase in serum SIRT3, one of the relevant oxidative–antioxidant balance parameters, suggesting that systemic antioxidant imbalance may affect male reproductive potential. Significant positive correlations between concentrations of SIRT3 and SIRT5 in both analyzed body fluids suggest synergistic action of these enzymes. Furthermore, the negative correlations between seminal plasma CLU vs. SIRT3 and SIRT5 concentrations suggest different modes of their action in the maintenance of the oxidative–antioxidant balance.

ROC curve and cluster analysis revealed that seminal plasma relative reactivity of CLU glycans with MAA and the value of MAA/SNA ratio, together with SIRT3 and SIRT5 concentrations, may constitute an additional set of markers differentiating infertile oligoasthenoteratozoospermic patients from normozoospermic, asthenoteratozoospermic and teratozoospermic men. However, considering the fact that in multinomial logistic regression analysis of both CLU reactivity with MAA and the values of MAA/SNA ratio, the value of Spearman’s rank correlation coefficient in the correlation between these two parameters indicated that the predictor collinearity assumption was not met here; thus, the potential diagnostic utility of these seminal plasma biomarkers should be treated with caution. The results of multinomial regression analysis indicated the significant differences between the N and OAT group for SIRT3 only as well as between the N and T group for SIRT3 and SIRT5, which additionally confirmed the potential diagnostic utility of these seminal plasma parameters. For blood serum, based on the results of the ROC curve and cluster analysis, relative reactivities of CLU glycans with SNA, and MAA, SIRT3 and FRAP concentrations may be useful in the differentiation of normozoospermic patients from those with sperm abnormalities. The results of multinomial logistic regression analysis showed that the SNA relative reactivity with CLU glycans was the parameter that significantly differentiated the normozoospermic group from AT, OAT and T groups, and FRAP concentrations significantly differed between N and AT groups, indicating the potential utility of these blood serum biomarkers for differentiation of infertile patients with sperm parameters disorders.

In this study, we examined and compared two biological fluids, looking for an additional male infertility biomarkers and exploring the associations between the expression of sialic acid on CLU glycans as well as selected oxidative stress parameters. Lack of a representative control group of men at reproductive age with proven fertility as well as lack of information on patient BMI, duration of infertility and diseases related to reduced fertility are the limitations of our study that make it impossible to verify whether the analyzed parameters may constitute as useful biomarkers of idiopathic male infertility. Nevertheless, according to the ROC curve and cluster analyses, several seminal plasma and blood serum parameters may be proposed by us as useful additional male infertility indicators. It seems to be surprising that some serum parameters were associated with disorders of sperm parameters. However, one should not draw hasty conclusions, and the observed dependencies should be checked in subsequent studies covering not only a larger number of patients, but also extending the spectrum of the analyzed oxidative stress parameters. To the best of our knowledge, this is the first study extensively concerning CLU sialylation alterations as well as changes in oxidative stress parameter expression in both human biological fluids: seminal plasma and blood serum. Lectin–ELISA used in this study enables to mimic the interactions between glycans and their endogenic ligands, including glycans availability, and therefore, the observed glycan–lectin reactions correspond to the in vivo processes, showing also the accessibility of sugar residues for ligands present in the human organism. Further studies based on lectin–glycan interaction, especially when such interactions will be analyzed simultaneously for multiple lectins and/or multiple glycoproteins, may shed new light on the molecular processes accompanying changes in glycoproteins glycosylation, analyzed in the context of male fertility disorders. An additional benefit from our research was to expand knowledge about potential associations between the parameters we examined, which may guide further research aimed at developing new therapeutic strategies of male infertility, especially caused by multiple overlapping factors.

## Figures and Tables

**Table 1:** The values of seminal plasma and blood serum parameters analyzed in groups of patients with fertility problems.

Parameter	AT n^PL^ = 22 n^S^ = 15 Median (IQR)	N n^PL^ = 43 n^S^ = 18 Median (IQR)	OAT n^PL^ = 29 n^S^ = 27 Median (IQR)	T n^PL^ = 38 n^S^ = 31 Median (IQR)
**SNA^PL^** **(AU)**	0.203 (0.148–0.244)	0.199 (0.132–0.296)	0.184 (0.133–0.299)	0.202 (0.114–0.266)
**MAA^PL^** **(AU)**	0.933 (0.226–1.680)	0.376 ^♦^ (0.177–1.348)	0.171 ^†^* (0.098–0.214)	0.740 (0.354–1.476)
**MAA^PL^/SNA^PL^**	4.455 (1.256–7.423)	1.731 (0.460–4.634)	0.756 ^†^* (0.377–1.513)	3.781 (1.436–8.082)
**SIRT3^PL^** **(ng/mL)**	10.90 ^†^^♦^ (9.23–12.32)	9.35 ^†^^♦^ (5.68–11.11)	2.11 (1.76–2.42)	2.64 (2.11–3.67)
**SIRT5^PL^** **(ng/mL)**	5.72 (4.72–8.31)	7.28 ^♦^ (1.67–7.97)	1.34 ^†^* (1.17–1.49)	6.89 (1.38–7.72)
**TAS^PL^** **(mM)**	1.79 (1.59–1.99)	1.71 (1.54–1.97)	1.70 (1.51–1.93)	1.72 (1.55–1.85)
**FRAP^PL^** **(mM)**	3.68 (3.27–4.14)	3.65 (2.85–4.37)	3.04 (2.45–3.81)	3.72 (2.93–4.40)
**SNA^S^** **(AU)**	0.412 (0.366–0.477)	0.881 ^†^*^♦^ (0.630–1.140)	0.495 (0.368–0.613)	0.428 (0.356–0.525)
**MAA^S^** **(AU)**	0.016 (0.002–0.074)	0.081 (0.054–0.106)	0.020 (0.002–0.086)	0.035 (0.004–0.072)
**MAA^S^/SNA^S^**	0.034 (0.005–0.152)	0.081 (0.064–0.122)	0.036 (0.005–0.167)	0.085 (0.012–0.159)
**SIRT3^S^** **(ng/mL)**	8.94 (6.58–19.15)	2.73 *^♦^ (1.61–7.35)	6.27 (5.54–15.07)	5.93 (4.29–11.53)
**SIRT5^S^** **(ng/mL)**	2.25 (2.06–3.22)	2.01 (1.39–2.53)	2.02 (1.84–4.11)	2.05 (1.61–3.27)
**TAS^S^** **(mM)**	1.37 (1.26–1.82)	1.54 (1.42–1.70)	1.40 (1.32–1.52)	1.38 (1.29–1.75)
**FRAP^S^** **(mM)**	1.20 (1.00–1.35)	1.49 * (1.43–1.59)	1.33 (1.10–1.55)	1.38 (1.22–1.56)

SNA^PL^—relative reactivity of seminal plasma CLU glycans with *Sambucus nigra* agglutinin; MAA^PL^—relative reactivity of seminal plasma CLU glycans with *Maackia amurensis* agglutinin; MAA^PL^/SNA^PL^—seminal plasma sialylation ratio; SIRT3^PL^—seminal plasma SIRT3 concentration; SIRT5^PL^—seminal plasma SIRT5 concentration; TAS^PL^—seminal plasma total antioxidant status; FRAP^PL^—seminal plasma ferric reducing antioxidant power; SNA^S^—relative reactivity of serum CLU glycans with *Sambucus nigra* agglutinin; MAA^S^—relative reactivity of serum CLU glycans with *Maackia amurensis* agglutinin; MAA^S^/SNA^S^—serum sialylation ratio; SIRT3^S^—serum SIRT3 concentration; SIRT5^S^—serum SIRT5 concentration; TAS^S^—serum total antioxidant status; FRAP^S^—serum ferric reducing antioxidant power. AT—asthenoteratozoospermia, N—normozoospermia, OAT—oligoasthenoteratozoospermia, T—teratozoospermia. n^PL^ and n^S^—number of seminal plasma and serum samples, respectively. Significant differences vs.: ^†^ T group, * AT group, ^♦^ OAT group. Taking into account the Bonferroni correction, a two-tailed *p* value of less than 0.008 was considered significant.

**Table 2:** Summary of the results of receiver operating characteristic (ROC) curves analysis for seminal plasma parameters.

Parameter	Compared Groups		AUC	AUC with 95% Confidence Interval	Cut Off Point	Sensitivity	Specificity	*p*
**SNA^PL^**	AT	vs. N	0.489	0.319–0.658	0.140	0.833	0.310	0.894
	OAT	vs. N	0.512	0.361–0.662	0.155	0.690	0.310	0.877
	T	vs. N	0.479	0.333–0.624	0.165	0.676	0.414	0.774
	AT	vs. T	0.504	0.342–0.667	0.12	0.889	0.265	0.961
	OAT	vs. T	0.487	0.341–0.633	0.155	0.414	0.735	0.859
	OAT	vs. AT	0.503	0.333–0.673	0.147	0.379	0.722	0.974
**MAA^PL^**	AT	vs. N	0.611	0.446–0.776	0.911	0.556	0.690	0.186
	OAT	vs. N	0.715	0.571–0.858	0.224	0.793	0.724	0.003
	T	vs. N	0.375	0.229–0.521	2.111	1.000	0.103	0.094
	AT	vs. T	0.484	0.303–0.666	0.226	0.278	0.912	0.867
	OAT	vs. T	0.890	0.802–0.979	0.224	0.793	0.912	0.000
	OAT	vs AT	0.883	0.786–0.980	0.214	0.759	0.944	0.000
**MAA^PL^/SNA^PL^**	AT	vs. N	0.632	0.473–0.792	0.837	1.000	0.345	0.104
	OAT	vs. N	0.671	0.531–0.811	1.545	0.793	0.552	0.017
	T	vs. N	0.639	0.495–0.782	3.285	0.588	0.724	0.058
	AT	vs. T	0.513	0.348–0.679	6.000	0.444	0.706	0.877
	OAT	vs. T	0.836	0.738–0.934	2.033	0.897	0.676	0.000
	OAT	vs. AT	0.837	0.724–0.950	0.824	0.552	1.000	0.000
**SIRT3^PL^**	AT	vs. N	0.624	0.472–0.776	10.859	0.636	0.649	0.110
	OAT	vs. N	0.951	0.905–0.996	2.500	0.821	0.946	0.000
	T	vs. N	0.887	0.810–0.965	4.645	1.000	0.757	0.000
	AT	vs. T	0.906	0.813–0.999	8.604	0.818	1.000	0.000
	OAT	vs. T	0.646	0.500–0.791	2.500	0.821	0.533	0.050
	OAT	vs. AT	0.948	0.882–1.000	7.985	1.000	0.818	0.000
**SIRT5^PL^**	AT	vs. N	0.491	0.330–0.653	5.840	0.324	0.455	0.917
	OAT	vs. N	0.812	0.698–0.926	2.084	0.964	0.730	0.000
	T	vs. N	0.543	0.409–0.677	7.728	0.778	0.351	0.527
	AT	vs. T	0.460	0.302–0.617	5.840	0.545	0.639	0.616
	OAT	vs. T	0.759	0.631–0.887	2.084	0.964	0.694	0.000
	OAT	vs. AT	0.916	0.829–1.000	2.084	0.964	0.818	0.000
**TAS^PL^**	AT	vs. N	0.559	0.409–0.708	1.56	0.909	0.324	0.440
	OAT	vs. N	0.523	0.370–0.676	1.98	0.875	0.235	0.765
	T	vs. N	0.455	0.316–0.594	1.62	0.694	0.382	0.525
	AT	vs. T	0.618	0.468–0.768	1.53	1.000	0.250	0.124
	OAT	vs. T	0.466	0.308–0.624	1.38	0.167	0.972	0.671
	OAT	vs. AT	0.580	0.412–0.747	1.52	0.292	1.000	0.352
**FRAP^PL^**	AT	vs. N	0.509	0.365–0.653	3.393	0.727	0.442	0.902
	OAT	vs. N	0.629	0.498–0.759	3.141	0.586	0.698	0.054
	T	vs. N	0.497	0.369–0.626	2.251	0.972	0.116	0.969
	AT	vs. T	0.495	0.344–0.646	3.393	0.727	0.417	0.948
	OAT	vs. T	0.641	0.505–0.778	3.678	0.724	0.528	0.043
	OAT	vs. AT	0.345	0.192–0.497	4.585	0.103	0.955	0.046

SNA^PL^—relative reactivity of seminal plasma CLU glycans with *Sambucus nigra* agglutinin; MAA^PL^—relative reactivity of seminal plasma CLU glycans with *Maackia amurensis* agglutinin; MAA^PL^/SNA^PL^—seminal plasma CLU sialylation ratio; SIRT3^PL^—seminal plasma SIRT3 concentration; SIRT5^PL^—seminal plasma SIRT5 concentration; TAS^PL^—seminal plasma total antioxidant status; FRAP^PL^—seminal plasma ferric reducing antioxidant power. AT—asthenoteratozoospermia, N—normozoospermia, OAT—oligoasthenoteratozoospermia, T—teratozoospermia. Area under the ROC curve (AUC) is given with 95% confidence interval. Data with AUC equal or greater than 0.715 are marked in grey. Based on the AUC, the clinical value of the laboratory test can be defined as: 0–0.5—zero, 0.5–0.7—limited, 0.7–0.9—moderate and >0.9—high. An AUC of ≥0.7, *p* < 0.05, was used as the criterion demonstrating the moderate clinical value of an examined parameter.

**Table 3:** Summary of receiver operating characteristic (ROC) curves for serum parameters.

Parameter	Compared Groups		AUC	AUC with 95% Confidence Interval	Cut Off Point	Sensitivity	Specificity	*p*
**SNA^S^**	AT	vs. N	0.892	0.774–1.000	0.609	0.867	0.813	0.000
	OAT	vs. N	0.837	0.703–0.971	0.694	0.889	0.688	0.000
	T	vs. N	0.872	0.748–0.996	0.591	0.871	0.813	0.000
	AT	vs. T	0.549	0.367–0.732	0.477	0.800	0.419	0.600
	OAT	vs. T	0.575	0.423–0.728	0.532	0.444	0.774	0.334
	OAT	vs. AT	0.615	0.436–0.794	0.437	0.667	0.667	0.208
**MAA^S^**	AT	vs. N	0.756	0.582–0.930	0.023	0.600	0.875	0.004
	OAT	vs. N	0.650	0.480–0.821	0.024	0.556	0.875	0.083
	T	vs. N	0.716	0.551–0.880	0.035	0.516	0.875	0.010
	AT	vs. T	0.544	0.364–0.724	0.023	0.600	0.613	0.632
	OAT	vs. T	0.481	0.327–0.636	0.024	0.556	0.581	0.814
	OAT	vs. AT	0.556	0.379–0.732	0.105	0.222	1.000	0.537
**MAA^S^/SNA^S^**	AT	vs. N	0.577	0.355–0.799	0.055	0.600	0.813	0.496
	OAT	vs. N	0.534	0.356–0.711	0.036	0.519	0.875	0.711
	T	vs. N	0.510	0.341–0.679	0.138	0.355	0.938	0.907
	AT	vs. T	0.546	0.372–0.720	0.055	0.600	0.548	0.602
	OAT	vs. T	0.514	0.363–0.666	0.036	0.519	0.581	0.853
	OAT	vs. AT	0.522	0.343–0.701	0.02	0.741	0.400	0.808
**SIRT3^S^**	AT	vs. N	0.830	0.690–0.969	4.979	1.000	0.611	0.000
	OAT	vs. N	0.761	0.605–0.918	5.000	1.000	0.611	0.001
	T	vs. N	0.699	0.535–0.863	3.647	0.871	0.611	0.018
	AT	vs. T	0.740	0.595–0.884	6.044	0.933	0.516	0.001
	OAT	vs. T	0.621	0.477–0.765	5.000	1.000	0.290	0.010
	OAT	vs. AT	0.607	0.433–0.782	6.010	0.444	0.933	0.229
**SIRT5^S^**	AT	vs. N	0.663	0.476–0.850	2.035	0.867	0.500	0.088
	OAT	vs. N	0.382	0.207–0.556	2.063	0.593	0.500	0.185
	T	vs. N	0.525	0.352–0.698	1.414	0.871	0.278	0.776
	AT	vs. T	0.647	0.492–0.803	1.798	1.000	0.387	0.064
	OAT	vs. T	0.418	0.269–0.566	2.019	0.556	0.548	0.277
	OAT	vs. AT	0.607	0.435–0.780	2.019	0.556	0.867	0.221
**TAS^S^**	AT	vs. N	0.637	0.427–0.847	1.38	0.600	0.833	0.200
	OAT	vs. N	0.687	0.527–0.848	1.42	0.630	0.722	0.022
	T	vs. N	0.639	0.477–0.801	1.38	0.581	0.833	0.094
	AT	vs. T	0.514	0.322–0.706	1.28	0.333	0.806	0.886
	OAT	vs. T	0.501	0.349–0.653	1.64	0.926	0.290	0.988
	OAT	vs. AT	0.516	0.312–0.720	1.30	0.852	0.333	0.878
**FRAP^S^**	AT	vs. N	0.780	0.606–0.953	1.352	0.800	0.778	0.002
	OAT	vs. N	0.644	0.477–0.811	1.429	0.667	0.778	0.090
	T	vs. N	0.618	0.453–0.784	1.422	0.581	0.778	0.162
	AT	vs. T	0.701	0.531–0.871	1.208	0.600	0.774	0.021
	OAT	vs. T	0.566	0.410–0.721	1.168	0.370	0.903	0.408
	OAT	vs. AT	0.620	0.444–0.795	1.372	0.481	0.800	0.181

SNA^S^—relative reactivity of serum CLU glycans with *Sambucus nigra* agglutinin; MAA^S^—relative reactivity of serum CLU glycans with *Maackia amurensis* agglutinin; MAA^S^/SNA^S^—serum sialylation ratio; SIRT3^S^—serum SIRT3 concentration; SIRT5S—serum SIRT5 concentration; TAS^S^—serum total antioxidant status; FRAP^S^—serum ferric reducing antioxidant power. AT—asthenoteratozoospermia, N—normozoospermia, OAT—oligoasthenoteratozoospermia, T—teratozoospermia. Area under the ROC curve (AUC) is given with 95% confidence interval. Data with AUC equal or greater than 0.701 are marked in grey. Based on the AUC, the clinical value of the laboratory test can be defined as: 0–0.5—zero, 0.5–0.7—limited, 0.7–0.9—moderate and >0.9—high. An AUC of ≥0.7, *p* < 0.05, was used as the criterion demonstrating the moderate clinical value of an examined parameter.

**Table 4:** The results of multinomial logistic regression analysis of selected seminal plasma parameters.

Predictor (Parameter)	Group	OR	Low 95% CI	High 95% CI	Wald Statistics	*p*
**MAA^PL^** **(AU)**	AT	1.597	0.616	4.139	0.927	0.336
**MAA^PL^/SNA^PL^**		0.947	0.838	1.070	0.773	0.379
**SIRT3^PL^ (ng/mL)**		1.091	0.883	1.348	0.651	0.420
**SIRT5^PL^ (ng/mL)**		1.019	0.811	1.279	0.026	0.873
**MAA^PL^** **(AU)**	OAT	1.966	0.093	41.407	0.189	0.664
**MAA^PL^/SNA^PL^**		1.106	0.620	1.971	0.116	0.733
**SIRT3^PL^ (ng/mL)**		0.236	0.093	0.603	9.104	0.003
**SIRT5^PL^ (ng/mL)**		0.908	0.386	2.138	0.049	0.826
**MAA^PL^** **(AU)**	T	3.565	0.335	37.896	1.111	0.292
**MAA^PL^/SNA^PL^**		1.038	0.703	1.532	0.035	0.851
**SIRT3^PL^ (ng/mL)**		0.234	0.104	0.529	12.211	<0.001
**SIRT5^PL^ (ng/mL)**		2.008	1.226	3.287	7.681	0.006

MAA^PL^—relative reactivity of seminal plasma CLU glycans with *Maackia amurensis* agglutinin; MAA^PL^/SNA^PL^—seminal plasma CLU sialylation ratio; SIRT3^PL^—seminal plasma SIRT3 concentration; SIRT5^PL^—seminal plasma SIRT5 concentration. AT—asthenoteratozoospermia, OAT—oligoasthenoteratozoospermia, T—teratozoospermia. OR—odds ratio, CI—confidence interval. *p* value of less than 0.05 was considered significant.

**Table 5:** The results of multinomial logistic regression analysis of selected serum parameters.

Predictor (Parameter)	Group	OR	Low 95% CI	High 95% CI	Wald Statistics	*p*
**SNA^S^** **(AU)**	AT	0.000	0.000	0.048	9.088	0.003
**MAA^S^** **(AU)**		2.225	0.000	6.175 × 10^10^	0.004	0.948
**SIRT3^S^ (ng/mL)**		1.084	0.894	1.316	0.677	0.411
**FRAP^S^ (mM)**		0.016	0.000	0.822	4.237	0.040
**SNA^S^** **(AU)**	OAT	0.001	0.000	0.171	7.239	0.007
**MAA^S^ (AU)**		155.638	0.000	2.225 × 10^11^	0.220	0.639
**SIRT3^S^ (ng/mL)**		1.101	0.909	1.334	0.967	0.326
**FRAP^S^ (mM)**		0.205	0.007	6.049	0.843	0.359
**SNA^S^** **(AU)**	T	0.000	0.000	0.051	10.019	0.002
**MAA^S^** **(AU)**		3.247	0.000	3.655 × 10^9^	0.012	0.912
**SIRT3^S^ (ng/mL)**		1.036	0.853	1.259	0.129	0.719
**FRAP^S^ (mM)**		0.212	0.007	6.068	0.821	0.365

SNA^S^—relative reactivity of serum CLU glycans with *Sambucus nigra* agglutinin; MAA^S^—relative reactivity of serum CLU glycans with *Maackia amurensis* agglutinin; SIRT3^S^—serum SIRT3 concentration; FRAP^S^—serum ferric reducing antioxidant power. AT—asthenoteratozoospermia, OAT—oligoasthenoteratozoospermia, T—teratozoospermia. OR—odds ratio, CI—confidence interval. *p* value of less than 0.05 was considered significant.

## Data Availability

All data needed to evaluate the conclusions in the article are present in the article. Additional data related to this study are available upon reasonable request from the corresponding author or first author.

## Supplementary Materials

The following supporting information can be downloaded at: https://www.mdpi.com/article/10.3390/ijms231810598/s1.

## Abbreviations

AGP	α1-acid glycoprotein
ApoJ	apolipoprotein J
AT	asthenoteratozoospermia
ATP	adenosine triphosphate
AUC	area under the curve
BMI	body mass index
BS	blood serum
BSA	bovine serum albumin
CAT	catalase
CaCl_2_	calcium dichloride
CLU	clusterin
CV	coefficients of variations
DNA	deoxyribonucleic acid
ELISA	Enzyme-Linked Immunosorbent Assay
Fe^2+^	ferrous iron
Fe^3+^	ferric ion
FeCl_3_ × 6H_2_O	ferric chloride hexahydrate
FeSO_4_	ferrous sulfate
FRAP	ferric reducing antioxidant power
FRAP^PL^	seminal plasma ferric reducing antioxidant power
FRAP^S^	serum ferric reducing antioxidant power
Gal	galactose
GPX	glutathione peroxidase
HCl	hydrochloric acid
IQR	interquartile range
Le^a^	Lewis^a^ oligosaccharide structure
Le^b^	Lewis^b^ oligosaccharide structure
Le^x^	Lewis^x^ oligosaccharide structure
Le^y^	Lewis^y^ oligosaccharide structure
MAA	*Maackia amurensis* agglutinin
MAA^PL^	relative reactivity of seminal plasma CLU glycans with *Maackia amurensis* agglutinin
MAA^PL^/SNA^PL^	seminal plasma sialylation ratio
MAA^S^	relative reactivity of serum CLU glycans with *Maackia amurensis* agglutinin
MAA^S^/SNA^S^	serum sialylation ratio
MgCl_2_ × 6H_2_O	magnesium dichloride hexahydrate
MnCl_2_ × 4H_2_O	manganese(II) chloride tetrahydrate
mRNA	messenger RNA
mtDNA	mitochondrial DNA
N	normozoospermia
NAD+	nicotinamide adenine dinucleotide
Neu5Ac/NANA	N-acetylneuraminic acid
OAT	oligoasthenoteratozoospermia
OS	oxidative stress
PGC-1α	peroxisome proliferator-activated receptor γ coactivator 1α
RNA	ribonucleic acid
ROC	receiver operating characteristics
ROS	reactive oxygen species
SA	sialic acid
SIRT3	sirtuin-3
SIRT3^PL^	seminal plasma SIRT3 concentration
SIRT3^S^	serum SIRT3 concentration
SIRT5	sirtuin-5
SIRT5^PL^	seminal plasma SIRT5 concentration
SIRT5^S^	serum SIRT5 concentration
SIRTs	sirtuins
SNA	*Sambucus nigra* agglutinin
SNA^PL^	relative reactivity of seminal plasma CLU glycans with *Sambucus nigra* agglutinin
SNA^S^	relative reactivity of serum CLU glycans with *Sambucus nigra* agglutinin
SOD	superoxidase dismutase
SP	seminal plasma
T	teratozoospermia
TAC	total antioxidant capacity
TAS	total antioxidant status
TAS^PL^	seminal plasma total antioxidant status
TAS^S^	serum total antioxidant status
TBS	Tris-buffered saline
TPTZ	2,4,6-tripyridyl-s-triazine
WHO	World Health Organization
