# Supplementary material for: The Association between Clusterin Sialylation Degree and Levels of Oxidative–Antioxidant Balance Markers in Seminal Plasmas and Blood Sera of Male Partners with Abnormal Sperm Parameters
Source: Int J Mol Sci. 2022 Sep 13;23(18):10598. doi: 10.3390/ijms231810598 (PMC9501354; doi:10.3390/ijms231810598)
Supplement: Supplementary file 1 [file ijms-23-10598-s001.zip › ijms-1863372-supplementary.pdf]

Table S1. The differences in the values of seminal plasma and blood serum parameters analyzed between groups of patients with fertility problems without Bonferroni correction.

Parameter	AT n^{PL}=22 n^{S}=15 median (IQR)	N n^{PL}=43 n^{S}=18 median (IQR)	OAT n^{PL}=29 n^{S}=27 median (IQR)	T n^{PL}=38 n^{S}=31 median (IQR)
**SNA^{PL} (AU)**	0.203 (0.148-0.244)	0.199 (0.132-0.296)	0.184 (0.133-0.299)	0.202 (0.114-0.266)
**MAA^{PL} (AU)**	0.933 (0.226-1.680)	$0.376^{\blacklozenge}$ (0.177-1.348)	$0.171^{\dagger *}$ (0.098-0.214)	0.740 (0.354-1.476)
**MAA^{PL}/SNA^{PL}**	4.455 (1.256-7.423)	$1.731^{\blacklozenge}$ (0.460-4.634)	$0.756^{\dagger *}$ (0.377-1.513)	3.781 (1.436-8.082)
**$SIRT3^{PL}$ (ng/mL)**	$10.90^{\dagger \blacklozenge}$ (9.23-12.32)	$9.35^{\dagger \blacklozenge}$ (5.68-11.11)	2.11 (1.76-2.42)	2.64 (2.11-3.67)
**$SIRT5^{PL}$ (ng/mL)**	5.72 (4.72-8.31)	$7.28^{\blacklozenge}$ (1.67-7.97)	$1.34^{\dagger *}$ (1.17-1.49)	6.89 (1.38-7.72)
**TAS^{PL} (mM)**	1.79 (1.59-1.99)	1.71 (1.54-1.97)	1.70 (1.51-1.93)	1.72 (1.55-1.85)
**$FRAP^{PL}$ (mM)**	3.68 (3.27-4.14)	3.65 (2.85-4.37)	3.04 (2.45-3.81)	3.72 (2.93-4.40)
**SNA^{S} (AU)**	0.412 (0.366-0.477)	$0.881^{\dagger * \blacklozenge}$ (0.630-1.140)	0.495 (0.368-0.613)	0.428 (0.356-0.525)
**MAA^{S} (AU)**	0.016 (0.002-0.074)	$0.081^{\dagger *}$ (0.054-0.106)	0.020 (0.002-0.086)	0.035 (0.004-0.072)
**MAA^{S}/SNA^{S}**	0.034 (0.005-0.152)	0.081 (0.064-0.122)	0.036 (0.005-0.167)	0.085 (0.012-0.159)
**$SIRT3^{S}$ (ng/mL)**	$8.94^{\dagger}$ (6.58-19.15)	$2.73^{\dagger * \blacklozenge}$ (1.61-7.35)	6.27 (5.54-15.07)	5.93 (4.29-11.53)
**$SIRT5^{S}$ (ng/mL)**	2.25 (2.06-3.22)	2.01 (1.39-2.53)	2.02 (1.84-4.11)	2.05 (1.61-3.27)
**TAS^{S} (mM)**	1.37 (1.26-1.82)	$1.54^{\blacklozenge}$ (1.42-1.70)	1.40 (1.32-1.52)	1.38 (1.29-1.75)
**$FRAP^{S}$**	$1.20^{\dagger}$	1.49^{*}	1.33	1.38

**(mM)**	(1.00-1.35)	(1.43-1.59)	(1.10-1.55)	(1.22-1.56)

SNA^{PL} - relative reactivity of seminal plasma CLU glycans with *Sambucus nigra* agglutinin; MAA^{PL} - relative reactivity of seminal plasma CLU glycans with *Maackia amurensis* agglutinin; MAA^{PL}/SNA^{PL} - seminal plasma sialylation ratio; $SIRT3^{PL}$ - seminal plasma SIRT3 concentration; $SIRT5^{PL}$ - seminal plasma SIRT5 concentration; TAS^{PL} - seminal plasma total antioxidant status; $FRAP^{PL}$ - seminal plasma ferric reducing antioxidant power; SNA^{S} - relative reactivity of serum CLU glycans with *Sambucus nigra* agglutinin; MAA^{S} - relative reactivity of serum CLU glycans with *Maackia amurensis* agglutinin; MAA^{S}/SNA^{S} - serum sialylation ratio; $SIRT3^{S}$ - serum SIRT3 concentration; $SIRT5^{S}$ - serum SIRT5 concentration; TAS^{S} - serum total antioxidant status; $FRAP^{S}$- serum ferric reducing antioxidant power. AT – asthenoteratozoospermia, N - normozoospermia, OAT - oligoasthenoteratozoospermia, T - teratozoospermia. n^{PL} and n^{S} – number of seminal plasma and serum samples, respectively. Significant differences versus: †T group, *AT group, $^{\blacklozenge}$OAT group. The two-tailed *p*-Value of less than 0.05 was considered significant.

Table S2. The results of receiver operating characteristic (ROC) curves analysis for seminal plasma parameters.

Parameter	Compared group (vs. the rest of the samples taken together)	AUC	mean AUC	AUC with 95% confidence interval	Cut off point	Sensitivity	Specificity	*p*
**SNA^{PL}**	AT	0.499	0.492	0.364-0.633	0.120	0.889	0.239	0.986
	N	0.484		0.357-0.611	0.164	0.414	0.654	0.808
	OAT	0.499		0.376-0.623	0.155	0.414	0.716	0.992
	T	0.486		0.367-0.605	0.179	0.618	0.461	0.816
**MAA^{PL}**	AT	0.662	0.670	0.532-0.791	0.911	0.556	0.750	0.015
	N	0.500		0.370-0.630	0.261	0.724	0.407	0.997
	OAT	0.826		0.739-0.913	0.214	0.759	0.852	0.000
	T	0.693		0.594-0.792	0.313	0.824	0.592	0.000
**MAA/SNA^{PL}**	AT	0.653	0.692	0.533-0.773	0.837	1.00	0.337	0.012
	N	0.527		0.396-0.657	0.631	0.345	0.802	0.690
	OAT	0.777		0.686-0.868	2.033	0.897	0.556	0.000
	T	0.678		0.574-0.782	2.301	0.676	0.671	0.001
**SIRT3^{PL}**	AT	0.809	0.786	0.709-0.908	9.230	0.773	0.800	0.000
	N	0.769		0.684-0.854	5.677	0.757	0.763	0.000
	OAT	0.847		0.775-0.919	2.500	0.821	0.809	0.000
	T	0.720		0.630-0.811	4.645	1.000	0.540	0.000
**SIRT5^{PL}**	AT	0.633	0.655	0.516-0.749	1.466	0.955	0.376	0.026
	N	0.617		0.510-0.725	5.594	0.730	0.581	0.033
	OAT	0.816		0.730-0.902	2.084	0.964	0.737	0.000
	T	0.555		0.440-0.670	5.249	0.694	0.540	0.352
**TAS^{PL}**	AT	0.587	0.538	0.465-0.708	1.53	1.000	0.245	0.161
	N	0.489		0.365-0.613	1.55	0.324	0.780	0.865
	OAT	0.514		0.376-0.652	1.50	0.250	0.891	0.840
	T	0.562		0.454-0.669	1.93	0.889	0.288	0.260
**FRAP^{PL}**	AT	0.544	0.567	0.424-0.663	3.393	0.727	0.481	0.473
	N	0.542		0.433-0.650	4.713	0.209	0.920	0.453
	OAT	0.639		0.524-0.754	3.141	0.586	0.693	0.018
	T	0.544		0.434-0.653	4.219	0.333	0.787	0.435

SNA^{PL} - relative reactivity of seminal plasma CLU glycans with *Sambucus nigra* agglutinin; MAA^{PL} - relative reactivity of seminal plasma CLU glycans with *Maackia amurensis* agglutinin; MAA^{PL}/SNA^{PL} - seminal plasma CLU sialylation ratio; SIRT3^{PL} - seminal plasma SIRT3 concentration; SIRT5^{PL} - seminal plasma SIRT5 concentration; TAS^{PL} - seminal plasma total antioxidant status; FRAP^{PL} - seminal plasma ferric reducing antioxidant power. AT – asthenoteratozoospermia, N – normozoospermia, OAT – oligoasthenoteratozoospermia, T – teratozoospermia. Area under the ROC curve (AUC) is given with 95% confidence interval. Data with AUC greater than 0.700 are marked in grey. Based on the AUC, the clinical value of laboratory test can be defined as: 0-0.5 - zero, 0.5-0.7 - limited, 0.7-0.9 - moderate and >0.9 - high. An AUC of ≥0.7, $p<0.05$, was used as the criterion demonstrating the moderate clinical value of an examined parameter.

Table S3. The results of receiver operating characteristic (ROC) curves analysis for serum parameters.

Parameter	Compared group (vs. the rest of the samples taken together)	AUC	mean AUC	AUC with 95% confidence interval	Cut off point	Sensitivity	Specificity	*p*
**SNA^S**	AT	0.647	0.653	0.506-0.789	0.477	0.800	0.554	0.041
	N	0.863		0.745-0.981	0.612	0.813	0.822	0.000
	OAT	0.478		0.349-0.608	0.437	0.667	0.435	0.745
	T	0.625		0.510-0.740	0.569	0.839	0.448	0.034
**MAA^S**	AT	0.594	0.592	0.448-0.739	0.023	0.600	0.622	0.203
	N	0.700		0.553-0.847	0.036	0.875	0.548	0.008
	OAT	0.516		0.377-0.655	0.024	0.556	0.613	0.821
	T	0.557		0.436-0.677	0.080	0.871	0.310	0.357
**MAA/SNA^S**	AT	0.544	0.514	0.388-0.701	0.055	0.600	0.581	0.581
	N	0.524		0.391-0.657	0.055	0.875	0.493	0.724
	OAT	0.510		0.371-0.650	0.036	0.519	0.629	0.883
	T	0.479		0.348-0.609	0.015	0.355	0.759	0.748
**SIRT3^S**	AT	0.714	0.656	0.596-0.832	6.044	0.933	0.526	0.000
	N	0.749		0.605-0.892	3.608	0.611	0.945	0.001
	OAT	0.607		0.484-0.730	5.000	1.000	0.328	0.088
	T	0.555		0.432-0.677	6.630	0.645	0.500	0.381
**SIRT5^S**	AT	0.637	0.561	0.516-0.758	2.035	0.867	0.513	0.028
	N	0.588		0.431-0.745	1.388	0.278	0.945	0.273
	OAT	0.452		0.323-0.581	2.019	0.556	0.609	0.467
	T	0.566		0.437-0.695	1.795	0.387	0.817	0.313
**TAS^S**	AT	0.544	0.572	0.359-0.729	1.28	0.333	0.842	0.642
	N	0.656		0.525-0.787	1.39	0.833	0.534	0.019
	OAT	0.549		0.427-0.672	1.64	0.926	0.297	0.423
	T	0.538		0.408-0.668	1.38	0.581	0.600	0.570
**FRAP^S**	AT	0.691	0.610	0.542-0.840	1.352	0.800	0.579	0.012
	N	0.661		0.530-0.792	1.431	0.778	0.658	0.016
	OAT	0.544		0.404-0.685	1.176	0.407	0.797	0.537
	T	0.544		0.425-0.664	1.170	0.903	0.300	0.468

SNA^S - relative reactivity of serum CLU glycans with *Sambucus nigra* agglutinin; MAA^S - relative reactivity of serum CLU glycans with *Maackia amurensis* agglutinin; MAA^S/SNA^S - serum sialylation ratio; SIRT3^S - serum SIRT3 concentration; SIRT5^S - serum SIRT5 concentration; TAS^S - serum total antioxidant status; FRAP^S - serum ferric reducing antioxidant power. AT – asthenoteratozoospermia, N – normozoospermia, OAT – oligoasthenoteratozoospermia, T – teratozoospermia. Area under the ROC curve (AUC) is given with 95% confidence interval. Data with AUC equal or greater than 0.700 are marked in grey. Based on the AUC, the clinical value of laboratory test can be defined as: 0–0.5 - zero, 0.5–0.7 - limited, 0.7–0.9 - moderate and >0.9 - high. An AUC of ≥0.7, p<0.05, was used as the criterion demonstrating the moderate clinical value of an examined parameter.

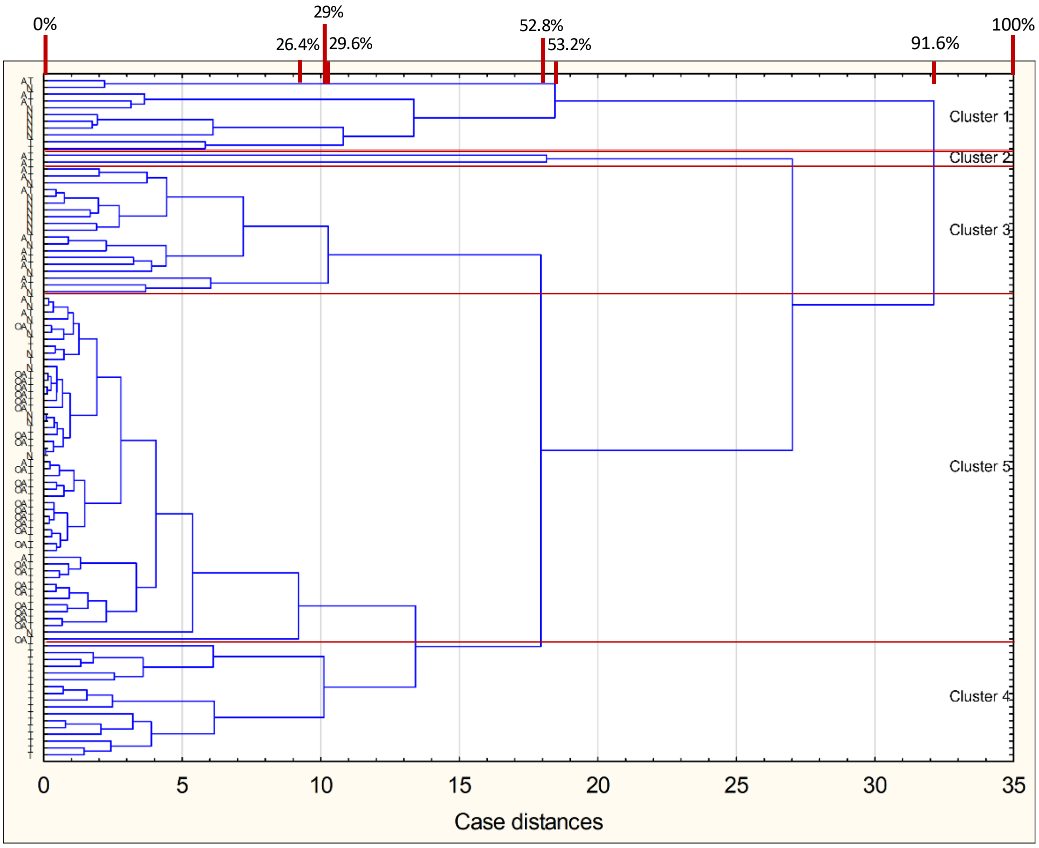

Figure S1. Dendrogram of cluster analysis of selected seminal plasma parameters. The cluster analysis was performed for parameters that simultaneously comply the following criteria: they allow for the differentiation of study groups and in the ROC curve analysis had moderate or high clinical value (AUC ≥0.715). Each seminal plasma sample is represented by a combination of four parameters: CLU relative reactivity with MAA, MAA/SNA ratio, SIRT3 and SIRT5 concentrations. AT – asthenoteratozoospermia, N – normozoospermia, OAT – oligoasthenoteratozoospermia, T – teratozoospermia. Case distances for each cluster were expressed in % of total distance (on x-axis 35=100%): cluster 1 – 53.2%, cluster 2 – 52.8%, cluster 3 – 29.6%, cluster 4 – 29%, cluster 5 – 26.4% (for details see Results section).

Table S4. The results of cluster analysis performed for seminal plasma samples.

**Cluster No.**	**AT (n=18)**	**N (n=25)**	**OAT (n=27)**	**T (n=30)**
	The number of samples (percentage participation in whole group)			
5 (n=51)	4 (22.2%)	9 (36%)	27 (100%)	11 (36.7%)
4 (n=17)	0 (0%)	0 (0%)	0 (0%)	17 (56.7%)
3 (n=19)	9 (50%)	10 (40%)	0 (0%)	0 (0%)
2 (n=2)	2 (11.1%)	0 (0%)	0 (0%)	0 (0%)
1 (n=11)	3 (16.7%)	6 (24%)	0 (0%)	2 (6.6%)

AT - asthenoteratozoospermia, N - normozoospermia, OAT - oligoasthenoteratozoospermia, T - teratozoospermia. Each group is presented as a vector of four parameters: relative reactivity of seminal plasma CLU glycans with *Maackia amurensis* agglutinin, CLU sialylation ratio (MAA^{PL}/SNA^{PL}), seminal plasma SIRT3 and SIRT5 concentrations.

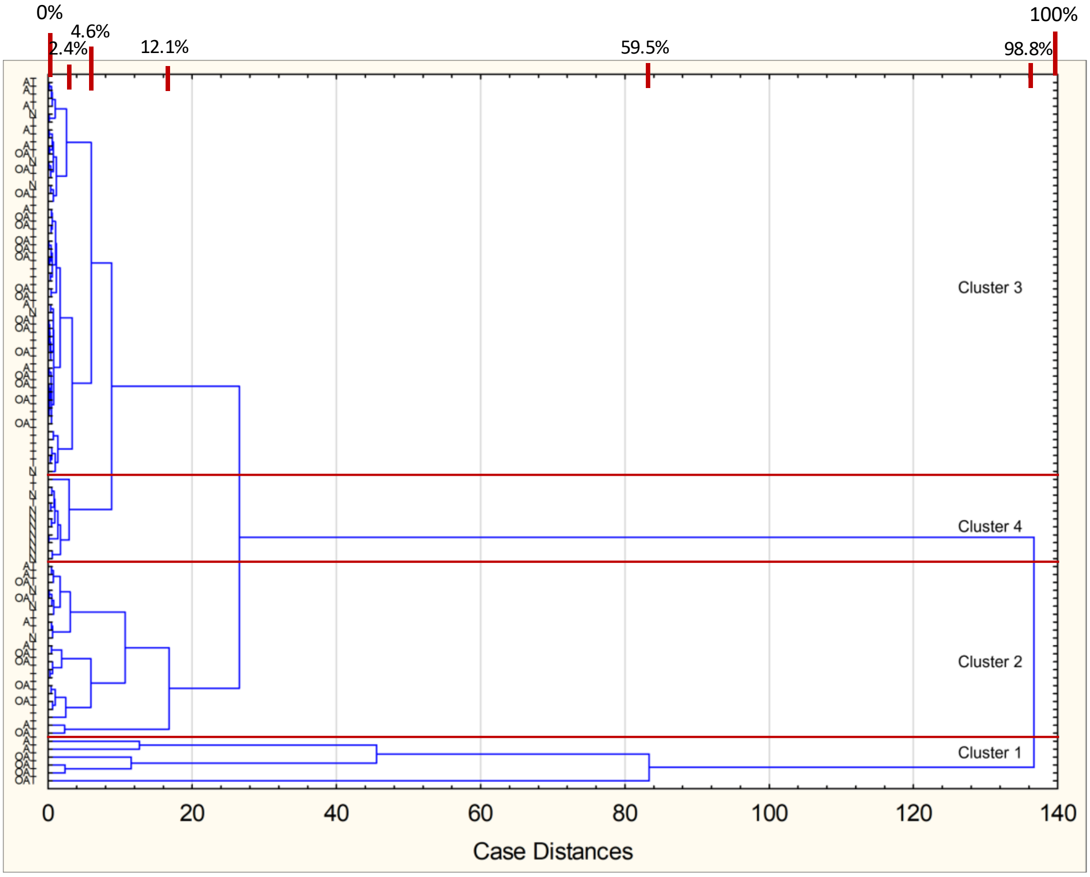

Figure S2. Dendrogram of cluster analysis of selected blood serum parameters. The cluster analysis was performed for parameters that simultaneously comply the following criteria: they allow for the differentiation of study groups and in the ROC curve analysis had moderate or high clinical value (AUC ≥0.701). Each seminal plasma sample is represented by a combination of four parameters: CLU relative reactivity with SNA and MAA, SIRT3 and FRAP concentrations. AT – asthenoteratozoospermia, N – normozoospermia, OAT – oligoasthenoteratozoospermia, T – teratozoospermia. Case distances for each cluster were expressed in % of total distance (on x-axis 140=100%): cluster 1 – 59.5%, cluster 2 – 12.1%, cluster 3 – 4.6%, cluster 4 – 2.3% (for details see Results section).

Table S5. The results of cluster analysis performed for blood serum samples.

**Cluster No.**	**AT (n=15)**	**N (n=16)**	**OAT (n=28)**	**T (n=30)**
	The number of samples (percentage participation in whole group)			
4 (n=11)	0 (0%)	8 (50%)	0 (0%)	3 (10%)
3 (n=50)	8 (53.4%)	5 (31.2%)	17 (60.7%)	20 (66.7%)
2 (n=22)	5 (33.3%)	3 (18.8%)	7 (25%)	7 (23.3%)
1 (n=6)	2 (13.3%)	0 (0%)	4 (14.3%)	0 (0%)

AT - asthenoteratozoospermia, N - normozoospermia, OAT - oligoasthenoteratozoospermia, T - teratozoospermia. Each group is presented as a vector of four parameters: relative reactivity of serum CLU glycans with *Sambucus nigra* agglutinin, relative reactivity of serum CLU glycans with *Maackia amurensis* agglutinin, serum SIRT3 and FRAP concentrations.
